# Molecular mechanisms of pain in acute pancreatitis: recent basic research advances and therapeutic implications

**DOI:** 10.3389/fnmol.2023.1331438

**Published:** 2023-12-22

**Authors:** Yongzi Wu, Chenxia Han, Rong Luo, Wenhao Cai, Qing Xia, Ruotian Jiang, Pawel E. Ferdek, Tingting Liu, Wei Huang

**Affiliations:** ^1^West China Centre of Excellence for Pancreatitis, Institute of Integrated Traditional Chinese and Western Medicine, West China-Liverpool Biomedical Research Centre, West China Hospital, Sichuan University, Chengdu, China; ^2^Laboratory of Anesthesia and Critical Care Medicine, National-Local Joint Engineering Research Center of Translational Medicine of Anesthesiology, West China Hospital, Sichuan University, Chengdu, China; ^3^Department of Cell Biology, Faculty of Biochemistry, Biophysics and Biotechnology, Jagiellonian University, Kraków, Poland; ^4^Institutes for Systems Genetics and Immunology and Inflammation, Frontiers Science Center for Disease-related Molecular Network, West China Hospital, Sichuan University, Chengdu, China; ^5^West China Biobank, West China Hospital, Sichuan University, Chengdu, China

**Keywords:** acute pancreatitis, visceral pain, neuropeptides, ion channels, endocannabinoid system

## Abstract

Although severe abdominal pain is the main symptom of acute pancreatitis, its mechanisms are poorly understood. An emerging body of literature evidence indicates that neurogenic inflammation might play a major role in modulating the perception of pain from the pancreas. Neurogenic inflammation is the result of a crosstalk between injured pancreatic tissue and activated neurons, which leads to an auto-amplification loop between inflammation and pain during the progression of acute pancreatitis. In this review, we summarize recent findings on the role of neuropeptides, ion channels, and the endocannabinoid system in acute pancreatitis-related pain. We also highlight potential therapeutic strategies that could be applied for managing severe pain in this disease.

## 1 Introduction

Acute pancreatitis (AP) is one of the most common gastrointestinal diseases that affects approximately 2.8 million people worldwide each year (Li et al., 2021) and shows an increasing global incidence (Iannuzzi et al., 2022). According to the latest epidemiological investigation, AP in the United States accounts for annual healthcare costs of $2.6 billion, while that for abdominal pain has increased to $9.5 billion (Peery et al., 2019). The characteristic abdominal pain has been adopted by the revised Atlanta classification as a diagnostic criterion for AP (Banks et al., 2013) and its intensity was associated with increased severity and mortality (Foldi et al., 2022). Opioid analgesics and non-steroidal anti-inflammatory drugs (NSAIDs) are commonly used for the management of pain in AP patients but are accompanied with side effects (Cai et al., 2021) and sometimes may even worsen the severity of AP (Barlass et al., 2018; Chen et al., 2022). Therefore, the development of new drugs and regimens for the treatment of pain in AP is required.

Visceral pain is mainly caused by factors such as tissue inflammation, ischemia, or dilation, all of which may damage internal organs and are often associated with patient’s emotional distress and reduced quality of life (Grundy et al., 2019). Gastrointestinal pain is a type of visceral pain commonly seen in many disorders including irritable bowel syndrome, appendicitis, and pancreatitis (Drewes et al., 2020). Early studies reported that certain factors, such as elevated pancreatic ductal and parenchymal pressure as well as duct obstruction, were associated with pain in pancreatitis (Karanjia et al., 1994; Dimagno, 1999). However, these factors alone cannot explain all the causes of pancreatic pain (Novis et al., 1985), nor do they provide a satisfactory explanation for the origin of the pain signals in the pancreas. Later, it was found that inflammatory cells were frequently gathered around the damaged nerves in pancreatic tissues from patients with chronic pancreatitis (Bockman et al., 1988), highlighting a potential role for the involvement of neurogenic inflammation in this setting. Inspired by this discovery, the focus of pain research in animal studies of AP has shifted from mechanical factors to neurogenic inflammation (Liddle and Nathan, 2004; Vera-Portocarrero and Westlund, 2005).

Neurogenic inflammation is the process in which activation of the primary afferent nerves gives rise to dorsal root reflexes in the spinal cord, and the primary nerve terminals release substances that induce inflammation in their target tissue (Willis, 1999). Noxious stimuli of peripheral tissues lead to the release or generation of multiple factors, such as growth factors, bradykinin, and hydrogen ions, which can activate the primary afferent neurons. Pathological activation of sensory neurons results in the release of neuropeptides including calcitonin-gene-related peptide (CGRP) and tachykinins such as substance P (SP), which can subsequently regulate the inflammatory response, plasma extravasation, and immune cell infiltration (Steinhoff et al., 2014). The resulting inflammation increases the excitability of the spinal cord, leading to an amplification of pain signals from the periphery. A similar crosstalk between injured pancreatic tissue and activated neurons creates an “auto-amplification loop” between inflammation and pain (neurogenic inflammation) during progression of AP (Liddle and Nathan, 2004; Vera-Portocarrero and Westlund, 2005). When this “auto-amplification loop” exceeds critical thresholds for unresolved systemic inflammatory response syndrome, irreversible persistent organ failure or multiple organ failure occurs, which significantly increases the risk of death (Barreto et al., 2021). Here, we attempt to collect and summarize current advances in pain research related to experimental AP, with the aim of pinpointing any research gaps that may warrant further exploration for potential translation.

## 2 Literature search

The literature search was carried out in multiple databases, such as PubMed, Web of Science, EMBASE, Science Citation Index Expanded, Cochrane Library, and Google Scholar using the MeSH terms “pancreatitis,” “acute pancreatitis,” “pancreatic acinar cells,” “pancreatic acini” combined with “pain” or “analgesia” as well as “analgesics” for basic research. All studies investigating pain in experimental AP were collated until May 2023. Reference lists of relevant reviews and other non-primary data sources related to this subject and returned by the search strategy were also manually screened. Only publications in English were included. The relevant articles were manually examined by at least two independent investigators.

## 3 Neuropeptides

### 3.1 Substance P and neurokinin receptors

Tachykinins are a family of neuropeptides that possess the COOH-terminal sequence (Phe-X-Gly-Leu-Met-NH_2_, X hydrophobic), including SP, neurokinin A, neurokinin B, neuropeptide K, and neuropeptide-γ (O’Connor et al., 2004). Tachykinins participate in important physiological and pathological processes such as epithelial secretion, inflammation, and nociception (O’Connor et al., 2004). SP, encoded by the *Tac1* gene (pre-pro-tachykinin-A, *Ppt-a*) (Steinhoff et al., 2014), is a well-known pro-inflammatory neuropeptide released from primary sensory nerve endings after the initial insult, and it modulates the perception of pain (Mashaghi et al., 2016; Hodo et al., 2020; Janket et al., 2023). SP binds to its endogenous receptors—neurokinin receptors (NKRs), with a high affinity for NK1R, but with a low affinity for NK2R and NK3R—to regulate release of various pro-inflammatory events (Nakanishi, 1991; Jensen et al., 2017). NK1R is widely expressed in neurons, immune cells, epithelial cells, and endothelial cells including pancreatic acinar cells (PACs) (Caberlotto et al., 2003).

#### 3.1.1 Expression and activation of SP/NK1R signaling pathway in pancreatic acinar cells

**FIGURE 1:**
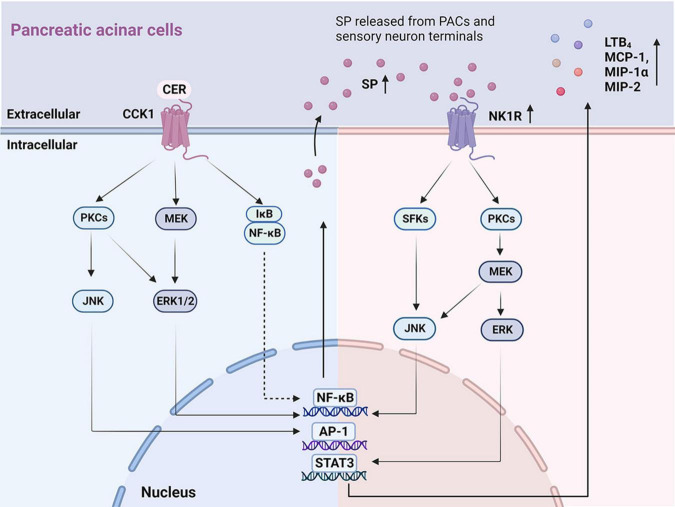
The key signaling pathways illustrating autocrine SP ⋅ and ⋅ SP/NK1R induced inflammatory mediators in PACs. On the **left:** Mechanisms of the autocrine effect of SP induced by CER in PACs. CER up-regulates SP and NK1R via the CCK1 receptor (Tamizhselvi et al., 2007; Koh et al., 2010, 2011a), activating signaling molecules that involve PKCs, JNK, MEK, ERK1/2, NF-κB, and AP-1. On the **right:** Mechanisms of SP binding to NK1R induced chemokine production in PACs. SP, released from PACs and sensory neuron terminals, binds to NK1R, regulating the production of inflammatory cytokines (LTB_4_, MCP-1, MIP-1α, and MIP-2) through the activation of SFKs, JNK, PKCs, MEK, ERK, NF-κB, AP-1, and STAT3. PACs, pancreatic acinar cells; SP, substance P; NK1R, neurokinin 1-receptor; CCK, cholecystokinin; CER, cerulein; PKC, protein kinase C; JNK, c-Jun N-terminal kinase; MEK, MAPK/ERK kinase; ERK 1/2, extracellular signal-regulated kinase 1/2; NF-κB, nuclear factor kappa-B; AP-1, activator protein-1; LBT_4_, leukotriene B_4_; MCP-1, monocyte chemotactic protein-1; MIP-1α, macrophage inflammatory protein-1α; MIP-2, macrophage inflammatory protein-2; SFKs, Src family kinases; STAT3, activator of transcription 3.

*In vitro* studies of SP and NK1R on PACs are summarized (Supplementary Table 1) with potential mechanism depicted in Figure 1. Both SP and NK1R were found in unstimulated PACs freshly isolated from rodents (Jensen et al., 1984; Patto et al., 1992; Sjödin and Gylfe, 1992). In AP patients, the expression levels of the SP and NK1R were greatly increased in the necrotic regions and adjacent inflamed regions of the pancreas, compared to normal controls (Han et al., 2021). Similar findings were observed in pancreatic tissues from the experimental model of AP in mice (Bhatia et al., 1998; Broccardo et al., 2006). Up-regulated *Nk1r* and *Ppt-a* mRNA expression and SP was found in isolated mouse PACs treated with cerulein (Koh et al., 2010), a cholecystokinin (CCK) analog commonly used to induce AP model via stimulating CCK1 receptor (Yang et al., 2020). An accumulation of literature evidence shows that several signaling molecules and pathways are involved in cerulein-induced SP/NK1R up-regulation in PACs, including mitogen-activated protein kinases (MAPKs), that is, c-Jun N-terminal kinase (JNK) and extracellular signal-regulated kinase 1/2 (ERK1/2), and other signaling molecules such as protein kinase C (PKC), activator protein-1 (AP-1), and nuclear factor kappa-B (NF-κB) (Koh et al., 2010, 2011a,b,^2012^). The inhibitors of CCK1 (Koh et al., 2010), MAPK (Koh et al., 2010, 2012), PKC (Koh et al., 2011a), and NF-κB (Ramnath and Bhatia, 2006; Koh et al., 2012) all suppressed cerulein-induced activation of SP/NK1R and reduced inflammation in the pancreas, suggesting that PKC-MAPK-NF-κB/AP-1 signaling pathway is crucial for cerulein-induced up-regulation of SP and NK1R. It was also reported that cerulein elevated the H_2_S levels in acinar cells; importantly NaHS (a H_2_S donor drug) caused an increase, while PAG (an inhibitor of H_2_S synthase cystathionine-γ-lyase)—a decrease in SP production, *Ppt-a* and *Nk1r* expression in cerulein-treated PACs (Tamizhselvi et al., 2007).

Upon binding of SP to NK1R located in the plasma membrane, the N-terminal domain of NK1R is phosphorylated, resulting in internalization of NK1R into the cytoplasm, which triggers downstream pro-inflammatory responses (Steinhoff et al., 2014). SP was found to cause increased synthesis of CC chemokines: monocyte chemotactic protein-1 (MCP-1) and macrophage inflammatory protein-1 alpha (MIP-1α), as well as a CXC chemokine: macrophage inflammatory protein-2 (MIP-2), in PACs (Ramnath and Bhatia, 2006). Furthermore, it was demonstrated that the signaling pathway SP/NK1R-Src family kinase (SFK)-MAPK-signal transducer and activator of transcription 3 (STAT3)/NF-κB/AP-1 was implicated in SP-mediated chemokine production (Ramnath and Bhatia, 2006; Ramnath et al., 2009). Pharmacological inhibition of NK1R (Ramnath et al., 2009; Koh et al., 2012; Han et al., 2021), SFK (Ramnath et al., 2009), or NF-κB (Ramnath and Bhatia, 2006; Koh et al., 2012) reduced SP-mediated up-regulation of chemokine production. Finally, the treatment of PACs with SP increased the production of leukotriene B_4_ (LTB_4_), a potent chemoattractant and inflammatory mediator, via PKCα/MAPK signaling pathway (Li et al., 2018).

These findings collectively indicate that the downstream SP/NK1R signaling pathways encompass the activation of SFK (Ramnath et al., 2009), PKC (Koh et al., 2011a), MAPK (Li et al., 2018), NF-κB, (Ramnath and Bhatia, 2006; Han et al., 2021), AP-1 (Ramnath et al., 2009), STAT3 (Ramnath et al., 2009) and induce even more generation of SP (Koh et al., 2012), increasing the expression of LTB_4_ (Li et al., 2018) and chemokine production (MCP-1, MIP-1α, and MIP-2) (Ramnath and Bhatia, 2006; Ramnath et al., 2009).

#### 3.1.2 Activation of the pancreatic acinar cells’ SP/NK1R signaling pathway promotes pancreatic inflammation

A number of studies have investigated the role of the SP/NK1R signaling pathway in the experimental models of AP in rodents (Supplementary Table 2). *In vivo* models of AP mainly include the following (Saloman et al., 2019; Yang et al., 2020): I. Secretagogue-induced hyperstimulation model, such as cerulein, which is an analog of cholecystokinin; II. Necrotizing AP model in rats or mice caused by specific amino acids, such as L-arginine, L-ornithine, and L-lysine; III. Alcohol-relevant AP model induced by a combination of ethanol and free fatty acids; IV. Surgical AP models induced by pancreatic duct infusion of sodium taurocholate (NaTC) or taurolithocholic acid 3-sulfate (TLCS), or simply by pancreatic duct ligation. Various AP animal models, including those mentioned above, have been explored in research related to AP-associated pain. An early study reported that SP induced plasma extravasation in the mouse gastrointestinal tract and pancreas, and this effect was abolished by administration of an NK1R antagonist (Figini et al., 1997). Importantly, the loss of NK1R markedly decreased the severity of cerulein-induced AP (CER-AP) in mice (Bhatia et al., 1998), a most commonly employed animal model, in which disease severity can be controlled by the dose and frequency of cerulein injections (Yang et al., 2020). Since NK1R expression increases in AP, but loss of NK1R has no direct effect on cerulein-induced secretion by PACs, the authors suggested that SP likely acts via NK1R on PACs, which then triggers the release of inflammatory mediators and increases the severity of AP (Bhatia et al., 1998). In accord with this, it was found that SP up-regulates LTB_4_ and results in acute lung injury through neutrophil reverse migration in a mouse model of AP induced by cerulein and lipopolysaccharide (CER/LPS-AP) (Li et al., 2018), which is associated with necrotizing inflammation and sepsis (Yang et al., 2020). What is more, neutral endopeptidase, an SP-degrading enzyme, was found to be expressed on PACs and to modulate peptide availability through binding to NK1R (Terashima et al., 1992). Caerulein was also shown to decrease neutral endopeptidase activity and its mRNA expression, leading to increased availability of SP and subsequent inflammation (Koh et al., 2011b). Genetic deletion or pharmacological inhibition of neutral endopeptidase exacerbated lung injury and elevated pancreatic myeloperoxidase both in CER-AP and in choline-deficient ethionine-supplemented diet-induced AP (CDE-AP), a severe necrotizing model caused by deranged amino acid metabolism in young and female mice (Maa et al., 2000; Koh et al., 2011b).

Deletion of NK1R reduced plasma extravasation, amylase, lipase as well as pancreatic neutrophil infiltration and necrosis in both CER-AP and CDE-AP models (Bhatia et al., 1998; Grady et al., 2000). It also decreased myeloperoxidase activity and pulmonary vascular permeability (Bhatia et al., 1998; Maa et al., 2000). Prophylactic and/or therapeutic administration of an NK1R antagonist (SR 14333, RP 67580, CP 96345, CP 99,994, SR 140333, or L 703,606) significantly reduced the severity of a number of experimental AP models (Grady et al., 2000; He et al., 2003; Lau et al., 2005; Lau and Bhatia, 2006; Sun and Bhatia, 2007; Camargo et al., 2008; Ramnath et al., 2009; Barreto et al., 2010; Li et al., 2018) and affected nociceptive behavior (Vera-Portocarrero and Westlund, 2004).

### 3.2 Calcitonin-gene-related peptide

Calcitonin-gene-related peptide is a 37 amino neuropeptide, which—similarly to SP—acts as an important pain mediator (Brain and Grant, 2004). It is released from the primary sensory nerves and is regulated by neurotransmitters and vasoactive substances, such as 5-hydroxytryptamine (5-HT), bradykinin, and nitric oxide (Brain and Grant, 2004). CGRP triggers vasodilatory effects, which have been shown to protect against ischemia-reperfusion injury (IRI) (Russell et al., 2014). CGRP binds to its receptors, composed of two subunits: (1) receptor activity modified protein 1 (RAMP1) and (2) calcitonin receptor-like receptor; the binding of CGRP induces an increase in cyclic adenosine monophosphate (cAMP), which is widely known to play critical roles in the regulation of metabolism, inflammation, and development of fibrosis in different tissues (Hay et al., 2018; Russo and Hay, 2023).

Previous work on the role of CGRP in experimental AP is summarized in a table (Supplementary Table 3). CGRP immunoreactivity has been detected in the pancreatic nerve innervating fibers (Sternini and Brecha, 1986). Stimulation of sensory fibers and administration of exogenous CGRP reduced pancreatic injury, while ablation of sensory nerves aggravated the severity in CER-AP and IRI-AP in rats (Warzecha et al., 2001; Dembiński et al., 2003). Treatment with CGRP before and during CER-AP induction was shown to have a protective effect, whereas CGRP aggravated pancreatic injury when it was applied after CER-AP had already been induced (Warzecha et al., 1997, 1999). In contrast, both prophylactic and therapeutic application of CGRP improved pancreatic microcirculation and morphological changes in a more severe necrotizing model induced by superimposing pancreatic duct infusion with bile acid on CER-AP in rats (Schneider et al., 2009). These discrepancies related to CGRP in AP models may be due to distinct dominant pathophysiological processes occurring in these models, with hypercirculation present in edematous pancreatitis and hypoperfusion present in necrotizing pancreatitis (Klar et al., 1994). Recently, genetic ablation of RAMP1 to block CGRP-induced downstream signaling was shown to worsen the severity of CER-AP in mice evidenced by the elevation of interleukin-beta (IL-1β), IL-6, and MIP-1α and reduced PAC proliferation, highlighting an essential role of CGRP in dampening the innate immune response (Jochheim et al., 2019).

Intrathecal administration of CGRP antagonist (CGRP8-37) significantly decreased the number of Fos-like immunoreactive nuclei at levels T9, T11, and L1 in L-Arginine-induced AP (ARG-AP) in rats, a commonly used necrotizing model induced by disturbed amino acid metabolism (Wick et al., 2006b). This finding was contrary to the vasodilatory and anti-inflammatory effects of CGRP, indicating that nociception is partially mediated by the release of CGRP in the ARG-AP and highlighting a complex role of this neuropeptide in experimental models of AP.

## 4 Ion channels

**FIGURE 2:**
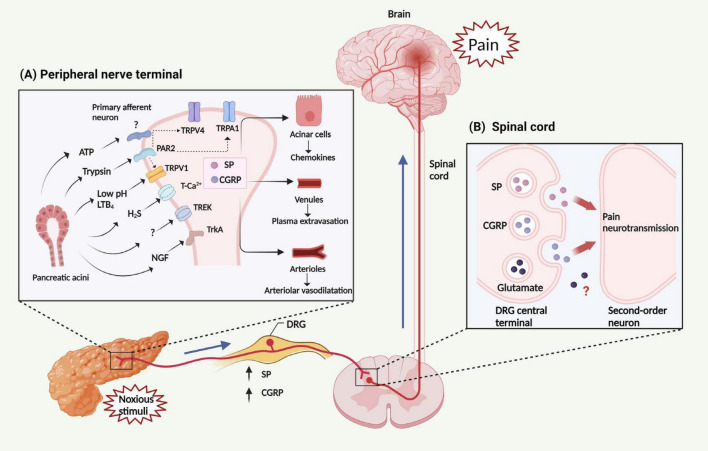
Mechanism and transmission of AP related-pain. **(A)** Noxious stimuli result in the generation and release of multiple factors from the pancreas, including trypsin, LTB_4_, H_2_S, ATP, NGF and protons (leading to a decrease in pH). These factors can activate several classes of pain-related receptors/ion channels expressed in peripheral nerve terminals, including members of the TRP family (TRPV1, TRPA1, and TRPV4), PAR2, TrkA, T-type Ca^2+^ channel, and TREK channel. The activation of PAR2 is able to *trans-*activate TRPV1, TRPA1, and TRPV4, subsequently triggering the activation of peripheral nerve terminals. Then, activated peripheral nerve terminals release neuropeptides within the pancreas, including SP and CGRP, leading to chemokine release from PACs, plasma extravasation from venules, and interstitial edema within arterioles. Collectively, these changes constitute neurogenic inflammation. **(B)** These factors stimulate primary sensory neurons, leading to the release of SP, CGRP and perhaps also glutamate from the DRG central terminal. These neuropeptides then activate receptors on spinal neurons, transmitting painful stimuli to the central nervous system. LTB_4_, leukotriene B_4_; ATP. adenosine triphosphate; NGF, nerve growth factor; TRP, Transient receptor potential channels; TRPV1, TRP vanilloid 1; TRPA1, TRP ankyrin 1; TRPV4, TRP vanilloid 4; PAR2, protease activated receptor 2; TrkA, tropomyosin receptor kinase A; TREK, TWIK-related K^+^ channel; SP, substance P; CGRP, calcitonin-gene-related peptide; PACs, pancreatic acinar cells; DRG, dorsal root ganglion.

Ion channels are plasma membrane proteins that regulate ion distribution, and mediate downstream signal events, which trigger a number of different cellular responses, including gene expression, muscle contraction, fertilization, cell division and cell death (Zaydman et al., 2012). Thus, abnormal ion channel expression or function may cause severe pathological conditions (Kasianowicz, 2012). In the pancreas, ion channels, particularly Ca^2+^ channels play a very important role, as they regulate the secretion of digestive proenzymes stored in the zymogen granules (Petersen et al., 2021). While physiological Ca^2+^ responses of PACs are primarily dependent on the Ca^2+^ channels present in the endoplasmic reticulum (inositol triphosphate receptors and ryanodine receptors) (Gerasimenko et al., 2014), other important ion channels in the pancreas include transient receptor potential channels (TRP), and K^+^ channels (Schnipper et al., 2020). TRP are a group of non-selective cation channels expressed on C and Aδ fibers of the primary sensory neurons (Benemei et al., 2015; Dai, 2016), which are primarily located in the plasma membrane and become activated by diverse nociceptive stimuli, including thermal and chemical factors (Figure 2A) (Zheng, 2013). Activation of TRP channels on the primary sensory neurons causes the release of neuropeptides such as SP and CGRP in the spinal cord to produce pain and in the pancreas to induce local inflammation (Figure 2B) (Rosenbaum et al., 2022). Among the TRP family, TRP vanilloid 1 (TRPV1), TRP ankyrin 1 (TRPA1), and TRP vanilloid 4 (TRPV4) have been studied in detail in the animal models of AP.

### 4.1 Transient receptor potential vanilloid 1

Transient receptor potential vanilloid 1 belongs to one of the most well-characterized TRP channels in experimental AP models, and some of the most relevant studies on TRPV1 are summarized in this review (Supplementary Table 4). It has been proposed that several different molecules could activate TRPV1 on the primary pancreatic innervating sensory neurons and promote neurogenic inflammation (Liddle, 2007). TRPV1 is a pH-sensitive cation channel, which can be activated in the acidic environment (Caterina and Julius, 2001). Injection of contrast solutions (pH 6.0/6.9), but not a solution of pH 7.3, caused a significant increase in serum amylase, pancreatic neutrophil infiltration and tissue damage, all of which were reverted by TRPV1 inhibition (Noble et al., 2008). Furthermore, proteinase-activated receptor-2 (PAR2), a G protein-coupled receptor (GPCR), is activated by trypsin and then it *trans-*activates TRPV1, thus being implicated in the processing of pancreatic pain (Nishimura et al., 2010). It was reported that LTB_4_ could act as an endogenous TRPV1 activator in pancreatic duct ligation-induced AP (PDL-AP) in rats (Vigna et al., 2011), a model which reflects the gallstone elicited etiology (Lerch and Aghdassi, 2010; Wan et al., 2012). A further study showed that LTB_4_ in PACs can bind to TRPV1 receptors on the pancreatic innervating sensory neurons and subsequently activate TRPV1, resulting in neurogenic inflammation (Shahid et al., 2015). In both CER-AP (Nathan et al., 2001) and ARG-AP (Wick et al., 2006a), it was shown that TRPV1 on the pancreatic sensory nerves was activated, which accompanied the release of CGRP and SP from both the pancreas and the dorsal horn to mediate neurogenic inflammation and pain, respectively. While genetic deletion of TRPV1 did not show a protective effect on CER-AP in mice (Romac et al., 2008), genetic deletion or pharmacological inhibition of TRPV1 has been consistently observed to reduce the severity of pancreatic inflammation, which was associated with the reduction of PAR2 activation, SP release, NK1R internalization, and 5-lipoxygenase expression as well as alleviated hyperalgesia in other experimental models of AP (Noble et al., 2006; Wick et al., 2006a; Nishimura et al., 2010; Vigna et al., 2011, 2014; Shahid et al., 2015). These findings provide compelling evidence that a cross-talk exists between PACs and sensory neurons.

### 4.2 Transient receptor potential ankyrin 1

There is a growing body of evidence suggesting that the TRPA1 channel plays a role in the perception of pain. This evidence comes in part from human subjects, and includes a gain-of-function point mutation, which was shown to be responsible for a familial episodic pain syndrome manifesting as episodes of severe upper body pain occurring after fasting or in response to physical stress (Kremeyer et al., 2010). In the pancreas, TRPA1 was shown to contribute to pain and inflammation in a mouse model of chronic pancreatitis induced by trinitrobenzene sulfonic acid (Cattaruzza et al., 2013). Recently this receptor was also implicated in Ca^2+^ signaling and cell death elicited by noxious stimuli in pancreatic stellate cells (Kusiak et al., 2022). The role of TRPA1 in experimental models of AP has been studied alone or simultaneously with TRPV1 (Supplementary Table 5). It was found that TRPA1 and TRPV1 synergistically attenuated pancreatic inflammation and pain in CER-AP in mice (Schwartz et al., 2011). Similarly, TRPA1 inhibition with a partial blockage of TRPV1 (Terada et al., 2013) or Cav3.2 (Terada et al., 2015), but not TRPA1 inhibition alone, reversed the hyperalgesia associated with CER-AP, further supporting the synergistic effect between TRPA1 and other ion channels in mediating pain. During the transition from acute to chronic pancreatitis, it was observed that the expression of TRPV1, TRPA1, and pERK in the pancreatic afferents was up-regulated; and this could be blocked by early (before week 3), but not late, intervention with TRPA1 and TRPV1 channel antagonists, leading to attenuated inflammation and pain-related behaviors (Schwartz et al., 2013).

### 4.3 Transient receptor potential vanilloid 4

The evidence for the TRPV4 channel in AP has also been gradually accumulating (Supplementary Table 6). *In vitro*, it was revealed that TRPV4 and Piezo1, the mechanosensitive ion channels, were expressed in PACs; Piezo1 activation triggered the opening of the TRPV4 channel, which resulted in a subsequent sustained elevation of intracellular Ca^2+^ release, mitochondrial depolarization, trypsin activation, and cell death in PACs (Swain et al., 2020). *In vivo*, Piezo1 or TRPV4 knockout mice exhibited protective effects against Piezo1 agonist- and pressure-induced AP (Swain et al., 2020). Similar to TRPV1 and TRPA1, there also appears to be a synergistic effect between TRPV4 and TRPA1 in mediating pain in AP. Literature reports suggest that both channels contribute to perception of pain in CER-AP in mice, but TRPV4 does not seem to mediate pancreatic inflammation (Ceppa et al., 2010). However, simultaneous inhibition of TRPV4 and TRPA1 significantly reduced pancreatic inflammation and pain in CER-AP in mice (Kanju et al., 2016).

### 4.4 Ca2^+^ and K^+^ channels

T-type Ca^2+^ channels have been shown to play a pivotal role in the processing of pain perception (Todorovic et al., 2001). It has also been reported in a hindpaw model in rats that activation of T-type Ca^2+^ channels is required in hyperalgesia induced by H_2_S, an endogenous product yielded from L-cysteine by cystathionine-γ-lyase (Kawabata et al., 2007). Initially, it was demonstrated that injection of NaHS into the pancreatic duct induced the expression of Fos protein in the superficial layers of the T8 and T9 spinal dorsal horn in rodents, which was suppressed by a T-type Ca^2+^ channel blocker (mibefradil) (Nishimura et al., 2009). Further, cystathionine-γ-lyase inhibitor or T-type Ca^2+^ channel blocker pretreatment alleviated allodynia/hyperalgesia, but had no effect on alleviating the severity of CER-AP in mice. In line with this, it was also found that the Cav3.2 T-type Ca^2+^ channel, targeted by H_2_S, participated in pain perception, whereas TRPA1 was down-regulated and played a secondary role in pancreatic nociceptive signaling in CER-AP in mice (Terada et al., 2015). These findings suggest that endogenous H_2_S-targeted T-type Ca^2+^ channels, which are likely expressed at the peripheral ending of the sensory nerves, contribute to pain resulting from AP. Additionally, blockers targeting the TRPA1 receptor and high-voltage gated calcium channel demonstrated an effective response profile in modulating nociception and the inflammatory process in the rat model of AP (Ricardo Carvalho et al., 2021).

TWIK-related K^+^ channel (TREK), a member of two-pore domain K^+^ channels, contains four transmembrane segments and two pore domains, is expressed in peripheral sensory neurons producing K^+^ currents which regulate the membrane potential of nociceptive neurons (Gada and Plant, 2019). A recent study showed that TREK-1/2 channels in sensory neurons were downregulated in CER-AP in mice and C3001a, a selective activator for TREK channels, reduced pancreatitis-related neurogenic inflammation and pain (Qiu et al., 2020).

## 5 Endocannabinoid system

The endocannabinoid system consists of cannabinoid endogenous ligands, their two receptors and metabolic enzymes. The endocannabinoid system plays a fundamental role in neurodevelopment (Bara et al., 2021) and has been implicated in a plethora of physiological and pathological processes (Di Marzo, 2018). While Δ9-tetrahydrocannabinol has been identified as the major psychotropic component of cannabis, N-arachidonoyl-ethanolamine (AEA) and 2-arachidonoylglycerol (2-AG) are the two best known endogenous cannabinoids among other structurally related signaling lipids that activate cannabinoid receptors (CBRs) (Di Marzo and Piscitelli, 2015). CBRs belong to a family of G protein coupled receptors (GPCRs) and include cannabinoid receptor 1 (CB1R), expressed in the central nervous system, and CB2R, which is present in the immune and peripheral nervous systems (Di Marzo and Piscitelli, 2015). Activation of CBRs plays a role in modulating pain, inflammation, cell growth, cell death, as well as diverse gastrointestinal functions such as intestinal motility and secretion (McKenna and McDougall, 2020; Brierley et al., 2023). It has been shown that cannabinoid-induced analgesia is largely mediated by CB1R in nociceptors (Agarwal et al., 2007).

In normal human pancreas, CB1R is expressed at a relatively low level, whereas the expression of CB2R is usually somewhat higher (Michalski et al., 2007). Analogously to the findings made in the human pancreas, both CB1R and CB2R have been detected in mouse PACs (Michalski et al., 2007; Linari et al., 2009; Huang et al., 2016). *In vitro* studies (Linari et al., 2009; Petrella et al., 2010; Cao et al., 2012; Huang et al., 2016; Xia et al., 2019) on CBR in freshly isolated PACs are summarized here (Supplementary Table 7). Of note is that while CBR agonists did not affect basal or carbachol- and cerulein-induced amylase secretion, a non-selective CBR agonist inhibited KCl-elicited elevation in amylase (Linari et al., 2009) as well as reduced cerulein-induced IL-6 and MCP-1 secretion in PACs (Petrella et al., 2010). Contrary to the above, a selective CB2R agonist inhibited Ca^2+^ oscillations in these cells induced by acetylcholine and L-arginine, but not by CCK (Huang et al., 2016). Furthermore, a range of CB2R agonists were shown to inhibit acetylcholine-induced and endocannabinoid-modulated Ca^2+^ oscillations (Xia et al., 2019). AP is initiated by elevated Ca^2+^ concentration in PACs, which in turn activates digestive pro-enzymes within the pancreas, leading to inflammation and necrosis (Petersen et al., 2021). Therefore, reducing Ca^2+^ influx into PACs and thereby alleviating cell damage is important for the treatment of AP.

In AP patients, prominent immunostaining for CB1R correlated with PAC necrosis, and there was a moderate increase in CB2R expression in PACs, ducts, and nerves (Michalski et al., 2007). Parallel to the changes in CB1R levels during AP, the pancreatic concentrations of AEA were dramatically increased compared to the normal human pancreas, but AG levels were not significantly different (Michalski et al., 2007). *In vivo* studies (Matsuda et al., 2005; Dembinski et al., 2006, 2008; Michalski et al., 2007; Linari et al., 2009; Zyromski et al., 2009; Petrella et al., 2010; Michler et al., 2013; Huang et al., 2016; Da Silva-Leite et al., 2018) on CBRs in experimental models of AP are listed here (Supplementary Table 8). In severe cases of CER-AP in mice, associated with necrotizing inflammation of the pancreas, the expression levels of both pancreatic CB1R and CB2R were up-regulated (Michalski et al., 2007); whereas, in mild and predominantly edematous CER-AP in rats, the pancreatic CB2R was down-regulated and CB1R remained unchanged (Linari et al., 2009). It was reported that a non-selective CBR agonist and CB2R agonists ameliorated the severity of mouse AP models (Michler et al., 2013). Furthermore, total polysaccharide of Ximenia americana (TPL-Xa) has been shown to inhibit hyper-nociception and inflammation, an effect abolished by CB2R antagonist but not CB1R antagonist, in CER-AP mice (Da Silva-Leite et al., 2018). These findings suggest that CB2R agonist and TPL-Xa protected against AP via activation of CB2R, highlighting its important role in the progression of the disease. Unlike CB2R, activation of CB1R has been shown to exacerbate the severity of CER-AP (Dembinski et al., 2006), while inhibition reduced the severity of sodium taurocholate-induced AP (NaTC-AP) in rats (Matsuda et al., 2005), a severe biliary necrotizing model with a high mortality (Wan et al., 2012). Inhibition of CB1R has also been shown to reduce severity in CER-AP in mice fed with high fat diet (Zyromski et al., 2009), an AP model that is associated with marked pancreatic necrosis and systemic inflammation (Patel et al., 2015). Interestingly, pre-treatment with a CB1R agonist increased, while post-treatment alleviated the severity of CER-AP in rats (Dembinski et al., 2008); however, pre-treatment with a non-selective CBR agonist reduced the severity, whereas the post-treatment showed the opposite effects (Petrella et al., 2010).

All of the above findings suggest a significant capacity for the regulation of CBRs expression in the pancreas. CB2R agonists reduce the severity of experimental AP, whereas the impact of CB1R agonists on the AP severity depends on the disease stage. Consequently, it is crucial to consider the timing of administration in clinical studies. However, the exact mechanisms through which CBRs influence AP pain are still unclear and require further exploration.

## 6 Other important mediators of pain

The relationship between AP and damage-associated molecular patterns, including cell-free DNA, high mobility group box 1 (HMGB1) or histones, has been intensively investigated (Hoque et al., 2011; Kang et al., 2014a,b). A study suggested that macrophage-derived HMGB1 could act as a pain mediator in the early stages of AP in mice, via the receptor for advanced glycation end products and the C-X-C motif chemokine ligand 12 and receptor type 4 axis (Irie et al., 2017). Other mediators such as trypsin (Hoogerwerf et al., 2004; Ceppa et al., 2011),α2δ1 subunit of the N-type voltage-activated Ca^2+^ channels (Smiley et al., 2004), bradykinin (Takemura et al., 2011), nerve growth factor (Toma et al., 2000) and tropomyosin receptor kinase A (Winston et al., 2003) have also been reported to affect disease severity and pain in experimental AP. Moreover, 5-HT and IL-6 are also important mediators of pain. Sumatriptan suppressed visceral pain of dibutyltin dichloride (DBTC) induced AP model through peripheral 5-HT B/D receptors (Vera-Portocarrero et al., 2008). IL-6 receptor antagonist also has been shown effective in DBTC model of pancreatitis (Vardanyan et al., 2010). Additionally, adenosine triphosphate (ATP) and glutamate serve as crucial excitatory neurotransmitters in the central nervous system (CNS) (Lieb and Forsmark, 2009; Shi et al., 2023). However, within the context of visceral pain, ATP acts as a stimulant, activating peripheral nerves and modulating afferent sensitivity (Grundy et al., 2019; Drewes et al., 2020). Glutamate, released by some axons extending into the dorsal horn, regulates secondary neurons involved in pain transmission (Lieb and Forsmark, 2009). Nevertheless, its precise role in AP remains unclear.

Opioids are widely used analgesics in clinical settings, and they constitute one of the primary treatments for pain in AP patients. A study demonstrated that dynorphin effectively alleviated pancreatitis pain by activating bradykinin B2 receptor (Chen et al., 2010). However, in animal experiments, researchers tend to focus more on the influence of opioids on the progression of AP, demonstrating controversial findings. Several studies have shown that fentanyl reduced the severity of SAP (Bálint et al., 2022), alleviated intestinal mucosal barrier damage through inhibiting MMP-9/FasL/Fas signaling pathway (Mo et al., 2022), and protected against heart injury by regulating NF-κB signaling pathway (Wang and Chen, 2017). On the other hand, morphine therapy worsened the severity of AP and impeded resolution and regeneration of the pancreas (Barlass et al., 2018), whereas another study indicated that morphine decreased vacuolization in edematous AP (Bálint et al., 2022). Additionally, the opioid blocker naltrexone did not affect the pancreatic pathology and the inflammatory response (Penido et al., 2012).

## 7 Mechanisms relevant to nociception and central nervous system

The central terminals of nociceptor sensory neurons form synapses with neurons in the dorsal horn of the spinal cord, transmitting processed information to the brain through local circuits. The presynaptic nociceptor terminals convey information about painful stimuli in the periphery, inflammation, and peripheral nerve damage to the postsynaptic neurons (Yekkirala et al., 2017). In terms of the pancreas, the innervation originates from dorsal root ganglion cells, with the majority of afferents in the visceral DRG belonging to the myelinated Aδ and unmyelinated C classes, both involved in nociception (Drewes et al., 2020). Pancreatic afferent nerves in DRG at T5-L2 send peripheral processes of these neurons projecting to the viscera through the celiac ganglion, while central processes travel through the dorsal roots to the spinal cord’s dorsal horn (Won et al., 1998; Candal et al., 2023; Münzberg et al., 2023). Both surgical celiac ganglionectomy and RTX therapy of the celiac ganglion inhibit primary sensory nerve signal transmission, resulting in a reduction of SP release in the pancreas (Noble et al., 2006). The spinal dorsal horn neurons in the laminae I–III exhibit enhanced release of SP and CGRP in L-arginine induced AP (Wick et al., 2006a). Thus, SP and CGRP are important molecules for pain transmission (Figure 2B). By means of signal transduction, these neurotransmitters induce central sensitization, a state of heightened neuronal activity and hyperexcitability in both the spinal cord and supraspinal regions (Ji et al., 2018; Shi and Wu, 2023). However, more studies are warranted to obtain a better understanding of the signaling mechanisms of pain transmission from peripheral neurons to the spinal cord and the changes of supraspinal regions in the context of AP.

## 8 Conclusion and perspectives

**FIGURE 3:**
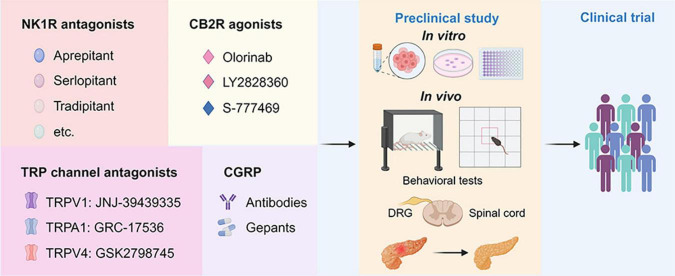
Drug discovery pipeline of potential agents for pharmaceutical treatment of AP-related pain. First, agents targeting NK1R, CB2R, TRP channels, and CGRP with translational potential are presented in the panels. Second, the promising agents will undergo comprehensive screening for efficacy and toxicity using *in vitro* assays and *in vivo* animal models for AP. This includes behavioral tests such as the von Frey test and open filed test to assess pain-related behaviors. Additionally, histological and biochemical parameters evaluating the severity of AP will be examined. Finally, one or two drugs with the best efficacy and safety profiles will advance to the clinical trial stage. NK1R, neurokinin 1-receptor; CGRP, calcitonin-gene-related peptide; TRPV1, transient receptor potential vanilloid 1; TRPA1, transient receptor potential ankyrin 1; TRPV4, transient receptor potential vanilloid 1; CB2R, cannabinoid receptor 2; DRG, dorsal root ganglion; PACs, pancreatic acinar cells.

There are opportunities for developing new analgesics and new therapeutic strategies targeting neuropeptides, ion channels, and G protein-coupled receptors (GPCRs) (Yekkirala et al., 2017). Neurogenic inflammation is an important mechanism of AP-associated pain, and ion channels, and neuropeptides are involved in this process. Furthermore, cannabinoid receptors, types of non-opioid GPCRs, are also important for the progression of AP. However, more research is needed to elucidate the communication of pain and the alterations in the spinal cord and central nervous system in the context of AP. The detailed knowledge of these mechanisms may provide several potential therapeutic targets for future drug research into AP-related pain (Figure 3).

In the realm of pain research, how to evaluate the pain in animal models is critical. Pain in AP patients is characterized by spontaneous, intense and persistent upper abdominal pain. Therefore, employing non-evoked behavioral assays become more clinically relevant for evaluating pain in animal models. In the future experimental studies, it is advisable to incorporate behavior assays, such as abdominal von Frey test and assessment of pain-related behaviors, to enhance the translational relevance of animal models for pain evaluation (Sadler et al., 2022). Abdominal von Frey test is performed by stimulating the upper abdomen of experimental animals with different strengths of filament to score pain behaviors, such as immediate escape, licking/scratching of the site stimulated with filaments, or strong retraction (Nishimura et al., 2010; Terada et al., 2015). Rodents’ pain-related behaviors can also be evaluated by analyzing the path information, distance traveled, hunching, and the length of time each mouse spent in the vertical plane or standing posture that required abdominal muscular stretch, a position considered to be painful in the presence of abdominal hypersensitivity (Schwartz et al., 2013).

As described above, animal studies show that NK1R antagonist significantly reduced pancreatic injury and pain. With the discovery of CP96345, the first non-peptide NK1R antagonist, researchers continued to explore clinically useful NK1R modulators and, as a result, aprepitant was introduced clinically for nausea and vomiting associated with anticancer chemotherapy (Huang and Korlipara, 2010). Subsequently, new applicants of NK1R antagonists have been continuously discovered, which includes antipruritics (Pojawa-Goła̧b et al., 2019), nausea, and vomiting in patients with idiopathic or diabetic gastroparesis (Carlin et al., 2021). Studies (Ramnath et al., 2009; Koh et al., 2012) have shown that CP96345 is effective in the treatment of experimental AP, and further clinical studies are needed to explore its therapeutic effect on AP. Apart from synthetic inhibitors, traditional Chinese medicine formula chaiqin chengqi decoction has been shown to ameliorate CER-AP and associated pain partially via inhibition of the SP/NK1R signaling pathway in PACs (Han et al., 2021). Furthermore, CGRP is also an important pain mediator in AP-related pain. Some monoclonal antibodies and compounds targeting CGRP or its receptor have been clinically approved for the treatment of migraine, as was elegantly summarized in a previous review article (Seidel et al., 2022), which have the potential to be tested in clinical trials. It was also reported that rutaecarpine, a major alkaloid component of the Chinese herb Wu-Zhu-Yu (Evodia rutaecarpa), showed protective effects against NaTC-AP in mice, via promoting the release of CGRP (Yan et al., 2018). Rutaecarpine was also found to inhibit tumor necrosis factor-α and IL-6 as well as upregulated IL-10 in CER/LPS-AP in mice (Huang et al., 2021).

Activation of neuronal terminals is a critical molecular event triggering neurogenic inflammation, so inhibition of ion channels or endogenous ligands on nerve terminals is crucial for reducing AP-related pain. Compounds targeting TRP channels also show significant potential for translation into the clinical practice (Koivisto et al., 2022). For example, mavatrep (JNJ-39439335), a TRPV1 antagonist, showed a marked reduction in pain induced by climbing stairs in patients with knee osteoarthritis (Manitpisitkul et al., 2018). GRC17536, a TRPA1 antagonist, has successfully gone through phase II clinical trials (Table 1), resulting in reduced pain scores in patients with painful diabetic polyneuropathy (Pallagi et al., 2022). However, none of the aforementioned inhibitors have been tested in AP models, emphasizing the potential importance of the proposed drug discovery pipeline (Figure 3). Nevertheless, some antagonists have been terminated or withdrawn from clinical trials because of lack of desired efficacy or side effects, including AZD1386 (Miller et al., 2014) and MK2295 (Eid, 2011) (Table 1).

**TABLE 1:** Potential therapeutic targets and agents for managing pain in AP.

Target	Mechanism	Agent	Indications	Phase	NCT number and status
Ion channels	TRPV1 antagonist	ABT-102	Healthy adults	I	NCT00854659, completed
		AZD1386	Esophageal pain	II	NCT01019928, completed
			Post-operative dental pain	II	NCT00672646, completed
			Peripheral neuropathic pain	II	NCT00976534, terminated
			Knee osteoarthritis	II	NCT00878501, terminated
		SB705498	Migraine	II	NCT00269022, completed
			Rectal pain	II	NCT00461682, terminated
			Dental pain after tooth extraction	II	NCT00281684, completed
		MK2295	Post-operative dental pain	II	NCT00387140, completed
		DWP-05195	Post-herpetic neuralgia	II	NCT01557010, completed
		Mavatrep (JNJ-39439335)	Knee osteoarthritis	I	NCT01343303, NCT00933582, completed
		SYL1001	Pain associated with dry eye syndrome	II	NCT02455999, completed
		NEO6860	Knee osteoarthritis	II	NCT02712957, completed
	TRPA1 antagonist	GRC 17536	Pain associated with diabetic peripheral neuropathy	II	NCT01726413, completed
Cannabinoid receptors	CB_2_R agonist	Olorinab (APD371)	Crohn’s disease	II	NCT03155945, completed
			Irritable bowel syndrome	II	NCT04043455, NCT04655599, terminated
		LY2828360	Osteoarthritic knee pain	II	NCT01319929, completed
		S-777469	Atopic dermatitis	II	NCT00703573, NCT00697710, completed
Voltage-gated calcium channels	Voltage-gated calcium channels inhibitor	Gabapentin	Neuropathy Pain	IV	NCT02074267, NCT00385671, completed
		Pregabalin	Pain associated with diabetic peripheral neuropathy	III	NCT00553475, NCT01057693, NCT01332149, completed
IL-6	IL-6 receptor inhibitor	Tocilizumab	Pain associated with hand osteoarthritis	III	NCT02477059, completed
NGF/TrkA signaling pathway	NGF antibody	Tanezumab	Osteoarthritis	III	NCT02709486, completed
			Cancer pain due to bone metastasis	III	NCT02609828, completed
		Fulranumab	Osteoarthritis	III	NCT02289716, completed
		Fasinumab	Lower back chronic pain	II/III	NCT02620020, completed
	TrkA antagonist	ONO-4474	Osteoarthritis	II	NCT02997696, terminated

TRPV1, transient receptor potential vanilloid 1; TRPA1, transient receptor potential ankyrin 1; TRPV4, transient receptor potential vanilloid 1; CB2R, cannabinoid receptor 2; IL-6, interleukin 6; NGF, nerve growth factor; TrkA, tropomyosin receptor kinase A.

The CBRs have also become potential targets for pain drugs (Aghazadeh Tabrizi et al., 2016). However, since CB1R is mainly expressed in the CNS, CB1R agonists are associated with side effects, whereas CB2R agonists produce analgesia in preclinical models without any major CNS side effects (Han et al., 2013). Preclinical studies in experimental AP have shown that CB2R agonists are able to reduce the disease severity (Michler et al., 2013; Huang et al., 2016). Therefore, CB2R agonists may be potential therapeutic agents for AP. Olorinab (APD371), a selective CB2R agonist, demonstrated mild to severe adverse events in clinical trials and improved abdominal pain scores in 14 patients with Crohn’s disease (Li et al., 2023). However, Olorinab has not been tested in experimental AP. Several other candidates are also currently evaluated in either phase I or II clinical trials (Aghazadeh Tabrizi et al., 2016). A detailed list can be found here (Table 1).

Moreover, voltage-gated calcium channel blockers, IL-6 small molecule inhibitor and NGF antibodies are also potential drugs for treating AP pain (Table 1). Gabapentin and pregabalin, the most commonly used treatment medicines targeting voltage-gated calcium channels, are beneficial in only a minority of individuals with hyperalgesia and neuropathic pain for unknown reasons (Yekkirala et al., 2017). Tocilizumab, an IL-6 small molecule inhibitor, demonstrated no greater efficacy than a placebo in relieving pain for patients with hand osteoarthritis (Richette et al., 2021). Notably, a recent phase III placebo-controlled trial revealed the potential of anti-NGF medications such as tanezumab to alleviate pain caused by bone metastases (Fallon et al., 2023). Another anti-NGF agents, Fasinumab, has shown promising results in improving both chronic low back pain and function (Dakin et al., 2021). Nevertheless, the effectiveness of these pharmaceutical agents in treating AP pain warrants further research.

## 9 Concluding remarks

In summary, we have provided an up-to-date review of the current research on pain associated with AP. Pain management in AP still represents a major clinical challenge and influences the clinical outcome of the disease. Understanding the pathophysiological mechanisms underlying pain in pancreatitis is a prerequisite toward designing new therapeutic approaches and improvement in the clinical practice.

## Author contributions

YW: Writing – original draft. CH: Funding acquisition, Writing – original draft. RL: Writing – review and editing. WC: Writing – review and editing. QX: Writing – review and editing. RJ: Writing – review and editing. PF: Funding acquisition, Writing – review and editing. TL: Supervision, Writing – review and editing. WH: Funding acquisition, Supervision, Writing – review and editing.

## References

[B1] Agarwal N. Pacher P. Tegeder I. Amaya F. Constantin C. E. Brenner G. J. (2007). Cannabinoids mediate analgesia largely via peripheral type 1 cannabinoid receptors in nociceptors. *Nat. Neurosci.* 10 870–879. 10.1038/nn1916 17558404 PMC2234438

[B2] Aghazadeh Tabrizi M. Baraldi P. G. Borea P. A. Varani K. (2016). Medicinal chemistry, pharmacology, and potential therapeutic benefits of cannabinoid CB2 receptor agonists. *Chem. Rev.* 116 519–560. 26741146 10.1021/acs.chemrev.5b00411

[B3] Bálint E. R. Für G. Kui B. Balla Z. Kormányos E. S. Orján E. M. (2022). Fentanyl but Not Morphine or Buprenorphine Improves the Severity of Necrotizing Acute Pancreatitis in Rats. *Int. J. Mol. Sci.* 23:1192. 10.3390/ijms23031192 PMC8835441 35163111

[B4] Banks P. A. Bollen T. L. Dervenis C. Gooszen H. G. Johnson C. D. Sarr M. G. (2013). Classification of acute pancreatitis–2012: Revision of the Atlanta classification and definitions by international consensus. *Gut* 62 102–111. 10.1136/gutjnl-2012-302779 23100216

[B5] Bara A. Ferland J. N. Rompala G. Szutorisz H. Hurd Y. L. (2021). Cannabis and synaptic reprogramming of the developing brain. *Nat. Rev. Neurosci.* 22 423–438. 10.1038/s41583-021-00465-5 34021274 PMC8445589

[B6] Barlass U. Dutta R. Cheema H. George J. Sareen A. Dixit A. (2018). Morphine worsens the severity and prevents pancreatic regeneration in mouse models of acute pancreatitis. *Gut* 67 600–602. 10.1136/gutjnl-2017-313717 28642332

[B7] Barreto S. G. Carati C. J. Schloithe A. C. Toouli J. Saccone G. T. P. (2010). The combination of neurokinin-1 and galanin receptor antagonists ameliorates caerulein-induced acute pancreatitis in mice. *Peptides* 31 315–321. 19944731 10.1016/j.peptides.2009.11.014

[B8] Barreto S. G. Habtezion A. Gukovskaya A. Lugea A. Jeon C. Yadav D. (2021). Critical thresholds: key to unlocking the door to the prevention and specific treatments for acute pancreatitis. *Gut* 70 194–203. 10.1136/gutjnl-2020-322163 32973069 PMC7816970

[B9] Benemei S. Patacchini R. Trevisani M. Geppetti P. (2015). TRP channels. *Curr. Opin. Pharmacol.* 22 18–23. 25725213 10.1016/j.coph.2015.02.006

[B10] Bhatia M. Saluja A. K. Hofbauer B. Frossard J. L. Lee H. S. Castagliuolo I. (1998). Role of substance P and the neurokinin 1 receptor in acute pancreatitis and pancreatitis-associated lung injury. *Proc. Natl. Acad. Sci. U. S. A.* 95 4760–4765. 9539812 10.1073/pnas.95.8.4760 PMC22564

[B11] Bockman D. E. Buchler M. Malfertheiner P. Beger H. G. (1988). Analysis of nerves in chronic pancreatitis. *Gastroenterology* 94 1459–1469. 3360267 10.1016/0016-5085(88)90687-7

[B12] Brain S. D. Grant A. D. (2004). Vascular actions of calcitonin gene-related peptide and adrenomedullin. *Physiol. Rev.* 84 903–934. 15269340 10.1152/physrev.00037.2003

[B13] Brierley S. M. Greenwood-Van Meerveld B. Sarnelli G. Sharkey K. A. Storr M. Tack J. (2023). Targeting the endocannabinoid system for the treatment of abdominal pain in irritable bowel syndrome. *Nat. Rev. Gastroenterol. Hepatol.* 20 5–25. 36168049 10.1038/s41575-022-00682-y

[B14] Broccardo M. Linari G. Agostini S. Amadoro G. Carpino F. Ciotti M. T. (2006). Expression of NK-1 and NK-3 tachykinin receptors in pancreatic acinar cells after acute experimental pancreatitis in rats. *Am. J. Physiol. Gastrointest. Liver Physiol.* 291 G518–G524. 10.1152/ajpgi.00505.2005 16782701

[B15] Caberlotto L. Hurd Y. L. Murdock P. Wahlin J. P. Melotto S. Corsi M. (2003). Neurokinin 1 receptor and relative abundance of the short and long isoforms in the human brain. *Eur. J. Neurosci.* 17 1736–1746. 10.1046/j.1460-9568.2003.02600.x 12752772

[B16] Cai W. Liu F. Wen Y. Han C. Prasad M. Xia Q. (2021). Pain management in acute pancreatitis: A systematic review and meta-analysis of randomised controlled trials. *Front. Med.* 8:782151. 10.3389/fmed.2021.782151 34977084 PMC8718672

[B17] Camargo E. A. Ferreira T. Ribela M. T. De Nucci G. Landucci E. C. Antunes E. (2008). Role of substance P and bradykinin in acute pancreatitis induced by secretory phospholipase A2. *Pancreas* 37 50–55. 10.1097/MPA.0b013e3185d9b9b 18580444

[B18] Candal R. Reddy V. Samra N. S. (2023). *Anatomy, Abdomen and Pelvis: Celiac Ganglia StatPearls.* Treasure Island FL: StatPearls Publishing. 30844156

[B19] Cao M. H. Li Y. Y. Xu J. Feng Y. J. Lin X. H. Li K. (2012). Cannabinoid HU210 protects isolated rat stomach against impairment caused by serum of rats with experimental acute pancreatitis. *PLoS One* 7:e52921. 10.1371/journal.pone.0052921 23285225 PMC3532296

[B20] Carlin J. L. Lieberman V. R. Dahal A. Keefe M. S. Xiao C. Birznieks G. (2021). Efficacy and safety of tradipitant in patients with diabetic and idiopathic gastroparesis in a randomized, placebo-controlled trial. *Gastroenterology* 160 76–87. 10.1053/j.gastro.2020.07.029 32693185

[B21] Caterina M. J. Julius D. (2001). The vanilloid receptor: a molecular gateway to the pain pathway. *Annu. Rev. Neurosci.* 24 487–517. 10.1146/annurev.neuro.24.1.487 11283319

[B22] Cattaruzza F. Johnson C. Leggit A. Grady E. Schenk A. K. Cevikbas F. (2013). Transient receptor potential ankyrin 1 mediates chronic pancreatitis pain in mice. *Am. J. Physiol. Gastrointest. Liver Physiol.* 304 G1002–G1012. 10.1152/ajpgi.00005.2013 23558009 PMC3680686

[B23] Ceppa E. Cattaruzza F. Lyo V. Amadesi S. Pelayo J. C. Poole D. P. (2010). Transient receptor potential ion channels V4 and A1 contribute to pancreatitis pain in mice. *Am. J. Physiol. Gastrointest. Liver Physiol.* 299 G556–G571. 10.1152/ajpgi.00433.2009 20539005 PMC2950679

[B24] Ceppa E. P. Lyo V. Grady E. F. Knecht W. Grahn S. Peterson A. (2011). Serine proteases mediate inflammatory pain in acute pancreatitis. *Am. J. Physiol. Gastrointest. Liver Physiol.* 300 G1033–G1042. 21436316 10.1152/ajpgi.00305.2010 PMC3774216

[B25] Chen Q. Vera-Portocarrero L. P. Ossipov M. H. Vardanyan M. Lai J. Porreca F. (2010). Attenuation of persistent experimental pancreatitis pain by a bradykinin b2 receptor antagonist. *Pancreas* 39 1220–1225. 10.1097/MPA.0b013e3181df1c90 20531238 PMC5690478

[B26] Chen Z. Jiang K. Liu F. Zhu P. Cai F. He Y. (2022). Safety and efficacy of intravenous hydromorphone patient-controlled analgesia versus intramuscular pethidine in acute pancreatitis: An open-label, randomized controlled trial. *Front. Pharmacol.* 13:962671. 10.3389/fphar.2022.962671 35991892 PMC9387897

[B27] Dakin P. Kivitz A. J. Gimbel J. S. Skrepnik N. DiMartino S. J. Emeremni C. A. (2021). Efficacy and safety of fasinumab in patients with chronic low back pain: A phase II/III randomised clinical trial. *Ann. Rheum. Dis.* 80, 509–517. 10.1136/annrheumdis-2020-217259 33199274 PMC7958114

[B28] Da Silva-Leite K. E. S. Girão D. De Freitas Pires A. Assreuy A. M. S. De Moraes P. A. F. Cunha A. P. (2018). Ximenia americana heteropolysaccharides ameliorate inflammation and visceral hypernociception in murine caerulein-induced acute pancreatitis: Involvement of CB2 receptors. *Biomed. Pharmacother.* 106 1317–1324. 10.1016/j.biopha.2018.07.067 30119202

[B29] Dai Y. (2016). TRPs and pain. *Semin. Immunopathol.* 38 277–291. 26374740 10.1007/s00281-015-0526-0

[B30] Dembinski A. Warzecha Z. Ceranowicz P. Dembinski M. Cieszkowski J. Pawlik W. W. (2006). Cannabinoids in acute gastric damage and pancreatitis. *J. Physiol. Pharmacol.* 57 137–154. 17218765

[B31] Dembiński A. Warzecha Z. Ceranowicz P. Jaworek J. Sendur R. Knafel A. (2003). Stimulation of sensory nerves and CGRP attenuate pancreatic damage in ischemia/reperfusion induced pancreatitis. *Med. Sci. Monit.* 9 Br418–Br425. 14646970

[B32] Dembinski A. Warzecha Z. Ceranowicz P. Warzecha A. M. Pawlik W. W. Dembinski M. (2008). Dual, time-dependent deleterious and protective effect of anandamide on the course of cerulein-induced acute pancreatitis. Role of sensory nerves. *Eur. J. Pharmacol.* 591 284–292. 10.1016/j.ejphar.2008.06.059 18593574

[B33] Di Marzo V. (2018). New approaches and challenges to targeting the endocannabinoid system. *Nat. Rev. Drug Discov.* 17 623–639. 30116049 10.1038/nrd.2018.115

[B34] Di Marzo V. Piscitelli F. (2015). The Endocannabinoid System and its Modulation by Phytocannabinoids. *Neurotherapeutics* 12 692–698. 26271952 10.1007/s13311-015-0374-6 PMC4604172

[B35] Dimagno E. P. (1999). Toward understanding (and management) of painful chronic pancreatitis. *Gastroenterology* 116 1252–1257. 10220520 10.1016/s0016-5085(99)70031-4

[B36] Drewes A. M. Olesen A. E. Farmer A. D. Szigethy E. Rebours V. Olesen S. S. (2020). Gastrointestinal pain. *Nat. Rev. Dis. Primers* 6:1. 10.1038/s41572-019-0135-7 31907359

[B37] Eid S. R. (2011). Therapeutic targeting of TRP channels–the TR(i)P to pain relief. *Curr. Top. Med. Chem.* 11 2118–2130. 10.2174/156802611796904898 21671881

[B38] Fallon M. Sopata M. Dragon E. Brown M. T. Viktrup L. West C. R. (2023). A randomized placebo-controlled trial of the anti-nerve growth factor antibody tanezumab in subjects with cancer pain due to bone metastasis. *Oncologist* 28, e1268–e1278. 10.1093/oncolo/oyad188 37343145 PMC10712717

[B39] Figini M. Emanueli C. Grady E. F. Kirkwood K. Payan D. G. Ansel J. (1997). Substance P and bradykinin stimulate plasma extravasation in the mouse gastrointestinal tract and pancreas. *Am. J. Physiol.* 272 G785–G793. 10.1152/ajpgi.1997.272.4.G785 9142909

[B40] Foldi M. Gede N. Kiss S. Vincze A. Bajor J. Szabo I. (2022). The characteristics and prognostic role of acute abdominal on-admission pain in acute pancreatitis: A prospective cohort analysis of 1432 cases. *Eur. J. Pain* 26 610–623. 10.1002/ejp.1885 34758174 PMC9299627

[B41] Gada K. Plant L. D. (2019). Two-pore domain potassium channels: emerging targets for novel analgesic drugs: IUPHAR Review 26. *Br. J. Pharmacol.* 176 256–266. 10.1111/bph.14518 30325008 PMC6295411

[B42] Gerasimenko J. V. Gerasimenko O. V. Petersen O. H. (2014). The role of Ca2+ in the pathophysiology of pancreatitis. *J. Physiol.* 592 269–280. 23897234 10.1113/jphysiol.2013.261784 PMC3922492

[B43] Grady E. F. Yoshimi S. K. Maa J. Valeroso D. Vartanian R. K. Rahim S. (2000). Substance P mediates inflammatory oedema in acute pancreatitis via activation of the neurokinin-1 receptor in rats and mice. *Br. J. Pharmacol.* 130 505–512. 10.1038/sj.bjp.0703343 10821777 PMC1572103

[B44] Grundy L. Erickson A. Brierley S. M. (2019). Visceral pain. *Annu. Rev. Physiol.* 81 261–284. 30379615 10.1146/annurev-physiol-020518-114525

[B45] Han C. Du D. Wen Y. Li J. Wang R. Jin T. (2021). Chaiqin chengqi decoction ameliorates acute pancreatitis in mice via inhibition of neuron activation-mediated acinar cell SP/NK1R signaling pathways. *J. Ethnopharmacol.* 274:114029. 10.1016/j.jep.2021.114029 33731310

[B46] Han S. Thatte J. Buzard D. J. Jones R. M. (2013). Therapeutic utility of cannabinoid receptor type 2 (CB(2) selective agonists. *J. Med. Chem.* 56 8224–8256. 23865723 10.1021/jm4005626

[B47] Hay D. L. Garelja M. L. Poyner D. R. Walker C. S. (2018). Update on the pharmacology of calcitonin/CGRP family of peptides: IUPHAR Review 25. *Br. J. Pharmacol.* 175 3–17. 10.1111/bph.14075 29059473 PMC5740251

[B48] He Z. J. Winston J. H. Yusuf T. E. Micci M. A. Elfert A. Xiao S. Y. (2003). Intraductal administration of an NK1 receptor antagonist attenuates the inflammatory response to retrograde infusion of radiological contrast in rats: implications for the pathogenesis and prevention of ERCP-induced pancreatitis. *Pancreas* 27 e13–e17. 10.1097/00006676-200307000-00018 12826913

[B49] Hodo T. W. De Aquino M. T. P. Shimamoto A. Shanker A. (2020). Critical neurotransmitters in the neuroimmune network. *Front. Immunol.* 11:1869. 10.3389/fimmu.2020.01869 32973771 PMC7472989

[B50] Hoogerwerf W. A. Shenoy M. Winston J. H. Xiao S. Y. He Z. Pasricha P. J. (2004). Trypsin mediates nociception via the proteinase-activated receptor 2: a potentially novel role in pancreatic pain. *Gastroenterology* 127 883–891. 10.1053/j.gastro.2004.07.002 15362043

[B51] Hoque R. Sohail M. Malik A. Sarwar S. Luo Y. Shah A. (2011). TLR9 and the NLRP3 inflammasome link acinar cell death with inflammation in acute pancreatitis. *Gastroenterology* 141 358–369. 10.1053/j.gastro.2011.03.041 21439959 PMC3129497

[B52] Huang H. Wang M. Guo Z. Wu D. Wang H. Jia Y. (2021). Rutaecarpine alleviates acute pancreatitis in mice and AR42J cells by suppressing the MAPK and NF-κB signaling pathways via calcitonin gene-related peptide. *Phytother. Res.* 35 6472–6485. 34661951 10.1002/ptr.7301

[B53] Huang S. C. Korlipara V. L. (2010). Neurokinin-1 receptor antagonists: a comprehensive patent survey. *Expert. Opin. Ther. Pat.* 20 1019–1045. 10.1517/13543776.2010.495121 20533894

[B54] Huang Z. Wang H. Wang J. Zhao M. Sun N. Sun F. (2016). Cannabinoid receptor subtype 2 (CB2R) agonist, GW405833 reduces agonist-induced Ca(2+) oscillations in mouse pancreatic acinar cells. *Sci. Rep.* 6:29757. 10.1038/srep29757 27432473 PMC4949433

[B55] Iannuzzi J. P. King J. A. Leong J. H. Quan J. Windsor J. W. Tanyingoh D. (2022). Global Incidence of Acute Pancreatitis Is Increasing Over Time: A Systematic Review and Meta-Analysis. *Gastroenterology* 162 122–134. 10.1053/j.gastro.2021.09.043 34571026

[B56] Irie Y. Tsubota M. Ishikura H. Sekiguchi F. Terada Y. Tsujiuchi T. (2017). Macrophage-derived HMGB1 as a pain mediator in the early stage of acute pancreatitis in mice: Targeting RAGE and CXCL12/CXCR4 Axis. *J. Neuroimmune Pharmacol.* 12 693–707. 10.1007/s11481-017-9757-2 28755135

[B57] Janket S. J. Fraser D. D. Baird A. E. Tamimi F. Sohaei D. Conte H. A. (2023). Tachykinins and the potential causal factors for post-COVID-19 condition. *Lancet Microbe* 4 e642–e650. 10.1016/S2666-5247(23)00111-8 37327802 PMC10263974

[B58] Jensen D. D. Lieu T. Halls M. L. Veldhuis N. A. Imlach W. L. Mai Q. N. (2017). Neurokinin 1 receptor signaling in endosomes mediates sustained nociception and is a viable therapeutic target for prolonged pain relief. *Sci. Transl. Med.* 9 eaal3447. 10.1126/scitranslmed.aal3447 28566424 PMC6034632

[B59] Jensen R. T. Jones S. W. Lu Y. A. Xu J. C. Folkers K. Gardner J. D. (1984). Interaction of substance P antagonists with substance P receptors on dispersed pancreatic acini. *Biochim. Biophys. Acta* 804 181–191. 6202326 10.1016/0167-4889(84)90148-4

[B60] Ji R. R. Nackley A. Huh Y. Terrando N. Maixner W. (2018). Neuroinflammation and central sensitization in chronic and widespread pain. *Anesthesiology* 129 343–366. 29462012 10.1097/ALN.0000000000002130 PMC6051899

[B61] Jochheim L. S. Odysseos G. Hidalgo-Sastre A. Zhong S. Staufer L. M. Kroiss M. (2019). The neuropeptide receptor subunit RAMP1 constrains the innate immune response during acute pancreatitis in mice. *Pancreatology* 19 541–547. 10.1016/j.pan.2019.05.455 31109903

[B62] Kang R. Lotze M. T. Zeh H. J. Billiar T. R. Tang D. (2014a). Cell death and DAMPs in acute pancreatitis. *Mol. Med.* 20 466–477. 25105302 10.2119/molmed.2014.00117 PMC4277549

[B63] Kang R. Zhang Q. Hou W. Yan Z. Chen R. Bonaroti J. (2014b). Intracellular Hmgb1 inhibits inflammatory nucleosome release and limits acute pancreatitis in mice. *Gastroenterology* 146 1097–1107. 10.1053/j.gastro.2013.12.015 24361123 PMC3965592

[B64] Kanju P. Chen Y. Lee W. Yeo M. Lee S. H. Romac J. (2016). Small molecule dual-inhibitors of TRPV4 and TRPA1 for attenuation of inflammation and pain. *Sci. Rep.* 6:26894. 10.1038/srep26894 27247148 PMC4887995

[B65] Karanjia N. D. Widdison A. L. Leung F. Alvarez C. Lutrin F. J. Reber H. A. (1994). Compartment syndrome in experimental chronic obstructive pancreatitis: effect of decompressing the main pancreatic duct. *Br. J. Surg.* 81 259–264. 10.1002/bjs.1800810236 8156353

[B66] Kasianowicz J. J. (2012). Introduction to ion channels and disease. *Chem. Rev.* 112 6215–6217. 24490587 10.1021/cr300444k

[B67] Kawabata A. Ishiki T. Nagasawa K. Yoshida S. Maeda Y. Takahashi T. (2007). Hydrogen sulfide as a novel nociceptive messenger. *Pain* 132 74–81. 17346888 10.1016/j.pain.2007.01.026

[B68] Klar E. Schratt W. Foitzik T. Buhr H. Herfarth C. Messmer K. (1994). Impact of microcirculatory flow pattern changes on the development of acute edematous and necrotizing pancreatitis in rabbit pancreas. *Dig. Dis. Sci.* 39 2639–2644. 10.1007/BF02087702 7995190

[B69] Koh Y. H. Moochhala S. Bhatia M. (2012). Activation of neurokinin-1 receptors up-regulates substance P and neurokinin-1 receptor expression in murine pancreatic acinar cells. *J. Cell. Mol. Med.* 16 1582–1592. 10.1111/j.1582-4934.2011.01475.x 22040127 PMC3823226

[B70] Koh Y.-H. Tamizhselvi R. Bhatia M. (2010). Extracellular signal-regulated kinase 1/2 and c-Jun NH2-terminal kinase, through nuclear factor-kappaB and activator protein-1, contribute to caerulein-induced expression of substance P and neurokinin-1 receptors in pancreatic acinar cells. *J. Pharmacol. Exp. Therap.* 332 940–948. 10.1124/jpet.109.160416 20007404

[B71] Koh Y.-H. Tamizhselvi R. Moochhala S. Bian J.-S. Bhatia M. (2011a). Role of protein kinase C in caerulein induced expression of substance P and neurokinin-1-receptors in murine pancreatic acinar cells. *J. Cell. Mol. Med.* 15 2139–2149. 10.1111/j.1582-4934.2010.01205.x 20973912 PMC4394224

[B72] Koh Y. H. Moochhala S. Bhatia M. (2011b). The role of neutral endopeptidase in caerulein-induced acute pancreatitis. *J. Immunol.* 187 5429–5439. 22013111 10.4049/jimmunol.1102011

[B73] Koivisto A. P. Belvisi M. G. Gaudet R. Szallasi A. (2022). Advances in TRP channel drug discovery: from target validation to clinical studies. *Nat. Rev. Drug Discov.* 21 41–59. 10.1038/s41573-021-00268-4 34526696 PMC8442523

[B74] Kremeyer B. Lopera F. Cox J. J. Momin A. Rugiero F. Marsh S. (2010). A gain-of-function mutation in TRPA1 causes familial episodic pain syndrome. *Neuron* 66 671–680. 20547126 10.1016/j.neuron.2010.04.030 PMC4769261

[B75] Kusiak A. A. Jakubowska M. A. Stopa K. B. Zhang X. Huang W. Gerasimenko J. V. (2022). Activation of pancreatic stellate cells attenuates intracellular Ca(2+) signals due to downregulation of TRPA1 and protects against cell death induced by alcohol metabolites. *Cell Death Dis.* 13:744. 10.1038/s41419-022-05186-w 36038551 PMC9421659

[B76] Lau H. Y. Bhatia M. (2006). The effect of CP96,345 on the expression of tachykinins and neurokinin receptors in acute pancreatitis. *J. Pathol.* 208 364–371. 16369913 10.1002/path.1899

[B77] Lau H. Y. Wong F. L. Bhatia M. (2005). A key role of neurokinin 1 receptors in acute pancreatitis and associated lung injury. *Biochem. Biophys. Res. Commun.* 327 509–515. 15629143 10.1016/j.bbrc.2004.12.030

[B78] Lerch M. M. Aghdassi A. A. (2010). The role of bile acids in gallstone-induced pancreatitis. *Gastroenterology* 138 429–433. 10.1053/j.gastro.2009.12.012 20034603

[B79] Li B. Han X. Ye X. Ni J. Wu J. Dai J. (2018). Substance P-regulated leukotriene B4 production promotes acute pancreatitis-associated lung injury through neutrophil reverse migration. *Int. Immunopharmacol.* 57 147–156. 10.1016/j.intimp.2018.02.017 29482159

[B80] Li C. L. Jiang M. Pan C. Q. Li J. Xu L. G. (2021). The global, regional, and national burden of acute pancreatitis in 204 countries and territories, 1990-2019. *BMC Gastroenterol.* 21:332. 10.1186/s12876-021-01906-2 34433418 PMC8390209

[B81] Li X. Chang H. Bouma J. De Paus L. V. Mukhopadhyay P. Paloczi J. (2023). Structural basis of selective cannabinoid CB(2) receptor activation. *Nat. Commun.* 14:1447. 10.1038/s41467-023-37112-9 PMC10017709 36922494

[B82] Liddle R. A. (2007). The role of Transient Receptor Potential Vanilloid 1 (TRPV1) channels in pancreatitis. *Biochim. Biophys. Acta* 1772 869–878. 17428642 10.1016/j.bbadis.2007.02.012 PMC1995747

[B83] Liddle R. A. Nathan J. D. (2004). Neurogenic inflammation and pancreatitis. *Pancreatology* 4 551–559. 15550764 10.1159/000082180

[B84] Lieb J. G. II Forsmark C. E. (2009). Review article: pain and chronic pancreatitis. *Aliment. Pharmacol. Ther.* 29 706–719. 19284407 10.1111/j.1365-2036.2009.03931.x

[B85] Linari G. Agostini S. Amadoro G. Ciotti M. T. Florenzano F. Improta G. (2009). Involvement of cannabinoid CB1- and CB2-receptors in the modulation of exocrine pancreatic secretion. *Pharmacol. Res.* 59 207–214. 10.1016/j.phrs.2008.11.002 19070664

[B86] Maa J. Grady E. F. Yoshimi S. K. Drasin T. E. Kim E. H. Hutter M. M. (2000). Substance P is a determinant of lethality in diet-induced hemorrhagic pancreatitis in mice. *Surgery* 128 232–239. 10.1067/msy.2000.107378 10922997

[B87] Manitpisitkul P. Flores C. M. Moyer J. A. Romano G. Shalayda K. Tatikola K. (2018). A multiple-dose double-blind randomized study to evaluate the safety, pharmacokinetics, pharmacodynamics and analgesic efficacy of the TRPV1 antagonist JNJ-39439335 (mavatrep). *Scand. J. Pain* 18 151–164. 10.1515/sjpain-2017-0184 29794306

[B88] Mashaghi A. Marmalidou A. Tehrani M. Grace P. M. Pothoulakis C. Dana R. (2016). Neuropeptide substance P and the immune response. *Cell Mol. Life Sci.* 73 4249–4264. 27314883 10.1007/s00018-016-2293-z PMC5056132

[B89] Matsuda K. Mikami Y. Takeda K. Fukuyama S. Egawa S. Sunamura M. (2005). The cannabinoid 1 receptor antagonist, AM251, prolongs the survival of rats with severe acute pancreatitis. *Tohoku J. Exp. Med.* 207 99–107. 16141678 10.1620/tjem.207.99

[B90] McKenna M. McDougall J. J. (2020). Cannabinoid control of neurogenic inflammation. *Br. J. Pharmacol.* 177 4386–4399. 33289534 10.1111/bph.15208 PMC7484507

[B91] Michalski C. W. Laukert T. Sauliunaite D. Pacher P. Bergmann F. Agarwal N. (2007). Cannabinoids ameliorate pain and reduce disease pathology in cerulein-induced acute pancreatitis. *Gastroenterology* 132 1968–1978. 10.1053/j.gastro.2007.02.035 17484889 PMC2268094

[B92] Michler T. Storr M. Kramer J. Ochs S. Malo A. Reu S. (2013). Activation of cannabinoid receptor 2 reduces inflammation in acute experimental pancreatitis via intra-acinar activation of p38 and MK2-dependent mechanisms. *Am. J. Physiol.* 304 G181–G192. 10.1152/ajpgi.00133.2012 23139224

[B93] Miller F. Björnsson M. Svensson O. Karlsten R. (2014). Experiences with an adaptive design for a dose-finding study in patients with osteoarthritis. *Contemp. Clin. Trials* 37 189–199. 10.1016/j.cct.2013.12.007 24394343

[B94] Mo Y. Zhang X. Lao Y. Wang B. Li X. Zheng Y. (2022). Fentanyl alleviates intestinal mucosal barrier damage in rats with severe acute pancreatitis by inhibiting the MMP-9/FasL/Fas pathway. *Immunopharmacol. Immunotoxicol.* 44 757–765. 10.1080/08923973.2022.2082304 35616237

[B95] Münzberg H. Berthoud H. R. Neuhuber W. L. (2023). Sensory spinal interoceptive pathways and energy balance regulation. *Mol. Metab.* 78:101817. 10.1016/j.molmet.2023.101817 PMC10590858 37806487

[B96] Nakanishi S. (1991). Mammalian tachykinin receptors. *Annu. Rev. Neurosci.* 14 123–136. 1851606 10.1146/annurev.ne.14.030191.001011

[B97] Nathan J. D. Patel A. A. Mcvey D. C. Thomas J. E. Prpic V. Vigna S. R. (2001). Capsaicin vanilloid receptor-1 mediates substance P release in experimental pancreatitis. *Am. J. Physiol. Gastrointest. Liver Physiol.* 281 G1322–G1328. 10.1152/ajpgi.2001.281.5.G1322 11668042

[B98] Nishimura S. Fukushima O. Ishikura H. Takahashi T. Matsunami M. Tsujiuchi T. (2009). Hydrogen sulfide as a novel mediator for pancreatic pain in rodents. *Gut* 58 762–770. 10.1136/gut.2008.151910 19201768

[B99] Nishimura S. Ishikura H. Matsunami M. Shinozaki Y. Sekiguchi F. Naruse M. (2010). The proteinase/proteinase-activated receptor-2/transient receptor potential vanilloid-1 cascade impacts pancreatic pain in mice. *Life Sci.* 87 643–650. 10.1016/j.lfs.2010.09.030 20932849

[B100] Noble M. D. Romac J. Vigna S. R. Liddle R. A. (2008). A pH-sensitive, neurogenic pathway mediates disease severity in a model of post-ERCP pancreatitis. *Gut* 57 1566–1571. 10.1136/gut.2008.148551 18625695 PMC4284069

[B101] Noble M. D. Romac J. Wang Y. Hsu J. Humphrey J. E. Liddle R. A. (2006). Local disruption of the celiac ganglion inhibits substance P release and ameliorates caerulein-induced pancreatitis in rats. *Am. J. Physiol. Gastrointest. Liver Physiol.* 291 G128–G134. 10.1152/ajpgi.00442.2005 16769810

[B102] Novis B. H. Bornman P. C. Girdwood A. W. Marks I. N. (1985). Endoscopic manometry of the pancreatic duct and sphincter zone in patients with chronic pancreatitis. *Dig. Dis. Sci.* 30 225–228. 3971834 10.1007/BF01347888

[B103] O’Connor T. M. O’connell J. O’brien D. I. Goode T. Bredin C. P. Shanahan F. (2004). The role of substance P in inflammatory disease. *J. Cell Physiol.* 201 167–180. 15334652 10.1002/jcp.20061

[B104] Pallagi P. Görög M. Papp N. Madácsy T. Varga Á Crul T. (2022). Bile acid- and ethanol-mediated activation of Orai1 damages pancreatic ductal secretion in acute pancreatitis. *J. Physiol.* 600 1631–1650. 10.1113/JP282203 35081662

[B105] Patel K. Trivedi R. N. Durgampudi C. Noel P. Cline R. A. Delany J. P. (2015). Lipolysis of visceral adipocyte triglyceride by pancreatic lipases converts mild acute pancreatitis to severe pancreatitis independent of necrosis and inflammation. *Am. J. Pathol.* 185 808–819. 10.1016/j.ajpath.2014.11.019 25579844 PMC4348470

[B106] Patto R. J. Vinayek R. Jensen R. T. Gardner J. D. (1992). Carbachol does not down-regulate substance P receptors in pancreatic acini. *Pancreas* 7 447–452. 1379366 10.1097/00006676-199207000-00005

[B107] Peery A. F. Crockett S. D. Murphy C. C. Lund J. L. Dellon E. S. Williams J. L. (2019). Burden and Cost of Gastrointestinal, Liver, and Pancreatic Diseases in the United States: Update 2018. *Gastroenterology* 156 254–272. 30315778 10.1053/j.gastro.2018.08.063 PMC6689327

[B108] Penido A. Coelho A. M. Molan N. T. Silva F. P. D’albuquerque L. A. Machado M. C. (2012). Do opioid receptors play a role in the pathogenesis of the inflammatory response in acute pancreatitis? *Acta Cir. Bras.* 27 600–605. 10.1590/s0102-86502012000900002 22936083

[B109] Petersen O. H. Gerasimenko J. V. Gerasimenko O. V. Gryshchenko O. Peng S. (2021). The roles of calcium and ATP in the physiology and pathology of the exocrine pancreas. *Physiol. Rev.* 101 1691–1744. 33949875 10.1152/physrev.00003.2021

[B110] Petrella C. Agostini S. Alema G. S. Casolini P. Carpino F. Giuli C. (2010). Cannabinoid agonist WIN55,212 in vitro inhibits interleukin-6 (IL-6) and monocyte chemo-attractant protein-1 (MCP-1) release by rat pancreatic acini and in vivo induces dual effects on the course of acute pancreatitis. *Neurogastroenterol. Motil.* 22 e1323. 10.1111/j.1365-2982.2010.01569.x 20659297

[B111] Pojawa-Goła̧b M. Jaworecka K. Reich A. (2019). NK-1 receptor antagonists and pruritus: Review of current literature. *Dermatol. Ther.* 9 391–405. 10.1007/s13555-019-0305-2 PMC6704190 31190215

[B112] Qiu Y. Huang L. Fu J. Han C. Fang J. Liao P. (2020). TREK Channel Family Activator with a Well-Defined Structure-Activation Relationship for Pain and Neurogenic Inflammation. *J. Med. Chem.* 63 3665–3677. 10.1021/acs.jmedchem.9b02163 32162512

[B113] Ramnath R. D. Bhatia M. (2006). Substance P treatment stimulates chemokine synthesis in pancreatic acinar cells via the activation of NF-kappaB. *Am. J. Physiol. Gastrointest. Liver Physiol.* 291 G1113–G1119. 16873895 10.1152/ajpgi.00177.2006

[B114] Ramnath R. D. Sun J. Bhatia M. (2009). Involvement of SRC family kinases in substance P-induced chemokine production in mouse pancreatic acinar cells and its significance in acute pancreatitis. *J Pharmacol Exp Ther* 329 418–428. 10.1124/jpet.108.148684 19211920 PMC2672875

[B115] Ricardo Carvalho V. P. Figueira Da Silva J. Buzelin M. A. Antônio Da Silva Júnior C. Carvalho Dos Santos D. Montijo Diniz D. (2021). Calcium channels blockers toxins attenuate abdominal hyperalgesia and inflammatory response associated with the cerulein-induced acute pancreatitis in rats. *Eur. J. Pharmacol.* 891:173672. 10.1016/j.ejphar.2020.173672 33190801

[B116] Richette P. Latourte A. Sellam J. Wendling D. Piperno M. Goupille P. (2021). Efficacy of tocilizumab in patients with hand osteoarthritis: Double blind, randomised, placebo-controlled, multicentre trial. *Ann. Rheum. Dis.* 80, 349–355. 10.1136/annrheumdis-2020-218547 33055078

[B117] Romac J. M. Mccall S. J. Humphrey J. E. Heo J. Liddle R. A. (2008). Pharmacologic disruption of TRPV1-expressing primary sensory neurons but not genetic deletion of TRPV1 protects mice against pancreatitis. *Pancreas* 36 394–401. 18437086 10.1097/MPA.0b013e318160222a

[B118] Rosenbaum T. Morales-Lázaro S. L. Islas L. D. (2022). TRP channels: a journey towards a molecular understanding of pain. *Nat. Rev. Neurosci.* 23 596–610. 10.1038/s41583-022-00611-7 35831443

[B119] Russell F. A. King R. Smillie S. J. Kodji X. Brain S. D. (2014). Calcitonin gene-related peptide: physiology and pathophysiology. *Physiol. Rev.* 94 1099–1142. 25287861 10.1152/physrev.00034.2013 PMC4187032

[B120] Russo A. F. Hay D. L. (2023). CGRP physiology, pharmacology, and therapeutic targets: migraine and beyond. *Physiol. Rev.* 103 1565–1644. 10.1152/physrev.00059.2021 36454715 PMC9988538

[B121] Sadler K. E. Mogil J. S. Stucky C. L. (2022). Innovations and advances in modelling and measuring pain in animals. *Nat. Rev. Neurosci.* 23 70–85. 34837072 10.1038/s41583-021-00536-7 PMC9098196

[B122] Saloman J. L. Albers K. M. Cruz-Monserrate Z. Davis B. M. Edderkaoui M. Eibl G. (2019). Animal models: challenges and opportunities to determine optimal experimental models of pancreatitis and pancreatic cancer. *Pancreas* 48 759–779. 10.1097/MPA.0000000000001335 31206467 PMC6581211

[B123] Schneider L. Hartwig W. Flemming T. Hackert T. Fortunato F. Heck M. (2009). Protective effects and anti-inflammatory pathways of exogenous calcitonin gene-related peptide in severe necrotizing pancreatitis. *Pancreatology* 9 662–669. 10.1159/000212099 19684430

[B124] Schnipper J. Dhennin-Duthille I. Ahidouch A. Ouadid-Ahidouch H. (2020). Ion Channel Signature in Healthy Pancreas and Pancreatic Ductal Adenocarcinoma. *Front. Pharmacol.* 11:568993. 10.3389/fphar.2020.568993 33178018 PMC7596276

[B125] Schwartz E. S. Christianson J. A. Chen X. La J. H. Davis B. M. Albers K. M. (2011). Synergistic role of TRPV1 and TRPA1 in pancreatic pain and inflammation. *Gastroenterology* 140 .e1281–.e1282. 10.1053/j.gastro.2010.12.033 21185837 PMC3066263

[B126] Schwartz E. S. La J.-H. Scheff N. N. Davis B. M. Albers K. M. Gebhart G. F. (2013). TRPV1 and TRPA1 antagonists prevent the transition of acute to chronic inflammation and pain in chronic pancreatitis. *J. Neurosci.* 33 5603–5611. 10.1523/JNEUROSCI.1806-12.2013 23536075 PMC3690366

[B127] Seidel M. F. Hügle T. Morlion B. Koltzenburg M. Chapman V. Maassenvandenbrink A. (2022). Neurogenic inflammation as a novel treatment target for chronic pain syndromes. *Exp. Neurol.* 356:114108. 10.1016/j.expneurol.2022.114108 35551902

[B128] Shahid R. A. Vigna S. R. Layne A. C. Romac J. M. J. Liddle R. A. (2015). Acinar Cell Production of Leukotriene B-4 Contributes to Development of Neurogenic Pancreatitis in Mice. *Cell. Mol. Gastroenterol. Hepatol.* 1 75–86. 10.1016/j.jcmgh.2014.11.002 25729765 PMC4339953

[B129] Shi Y. Gong C. Nan W. Zhou W. Lei Z. Zhou K. (2023). Intrathecal administration of botulinum toxin type a antagonizes neuropathic pain by countering increased vesicular nucleotide transporter expression in the spinal cord of chronic constriction injury of the sciatic nerve rats. *Neuropeptides* 100:102346. 10.1016/j.npep.2023.102346 37178626

[B130] Shi Y. Wu W. (2023). Multimodal non-invasive non-pharmacological therapies for chronic pain: mechanisms and progress. *BMC Med.* 21:372. 10.1186/s12916-023-03076-2 37775758 PMC10542257

[B131] Sjödin L. Gylfe E. (1992). A selective and potent antagonist of substance P receptors on pancreatic acinar cells. *Biochem. Int.* 27 145–153. 1378274

[B132] Smiley M. M. Lu Y. Vera-Portocarrero L. P. Zidan A. Westlund K. N. (2004). Intrathecal gabapentin enhances the analgesic effects of subtherapeutic dose morphine in a rat experimental pancreatitis model. *Anesthesiology* 101 759–765. 10.1097/00000542-200409000-00026 15329602 PMC2770328

[B133] Steinhoff M. S. Von Mentzer B. Geppetti P. Pothoulakis C. Bunnett N. W. (2014). Tachykinins and their receptors: contributions to physiological control and the mechanisms of disease. *Physiol. Rev.* 94 265–301. 10.1152/physrev.00031.2013 24382888 PMC3929113

[B134] Sternini C. Brecha N. (1986). Immunocytochemical identification of islet cells and nerve fibers containing calcitonin gene-related peptide-like immunoreactivity in the rat pancreas. *Gastroenterology* 90 1155–1163. 10.1016/0016-5085(86)90380-x 3082704

[B135] Sun J. Bhatia M. (2007). Blockade of neurokinin-1 receptor attenuates CC and CXC chemokine production in experimental acute pancreatitis and associated lung injury. *Am. J. Physiol.* 292 G143–G153. 10.1152/ajpgi.00271.2006 16873893

[B136] Swain S. M. Romac J. M. Shahid R. A. Pandol S. J. Liedtke W. Vigna S. R. (2020). TRPV4 channel opening mediates pressure-induced pancreatitis initiated by Piezo1 activation. *J. Clin. Invest.* 130 2527–2541. 10.1172/JCI134111 31999644 PMC7190979

[B137] Takemura Y. Furuta S. Hirayama S. Miyashita K. Imai S. Narita M. (2011). Upregulation of bradykinin receptors is implicated in the pain associated with caerulein-induced acute pancreatitis. *Synapse* 65 608–616. 10.1002/syn.20880 21484880

[B138] Tamizhselvi R. Moore P. K. Bhatia M. (2007). Hydrogen sulfide acts as a mediator of inflammation in acute pancreatitis: in vitro studies using isolated mouse pancreatic acinar cells. *J. Cell Mol. Med.* 11 315–326. 10.1111/j.1582-4934.2007.00024.x 17488480 PMC3822830

[B139] Terada Y. Fujimura M. Nishimura S. Tsubota M. Sekiguchi F. Kawabata A. (2015). Roles of Cav3.2 and TRPA1 channels targeted by hydrogen sulfide in pancreatic nociceptive processing in mice with or without acute pancreatitis. *J. Neurosci. Res.* 93 361–369. 25267397 10.1002/jnr.23490

[B140] Terada Y. Fujimura M. Nishimura S. Tsubota M. Sekiguchi F. Nishikawa H. (2013). Contribution of TRPA1 as a downstream signal of proteinase-activated receptor-2 to pancreatic pain. *J. Pharmacol. Sci.* 123 284–287. 10.1254/jphs.13128sc 24162021

[B141] Terashima H. Okamoto A. Menozzi D. Goetzl E. J. Bunnett N. W. (1992). Identification of neuropeptide-degrading enzymes in the pancreas. *Peptides* 13 741–748. 10.1016/0196-9781(92)90181-2 1359509

[B142] Todorovic S. M. Jevtovic-Todorovic V. Meyenburg A. Mennerick S. Perez-Reyes E. Romano C. (2001). Redox modulation of T-type calcium channels in rat peripheral nociceptors. *Neuron* 31 75–85. 10.1016/s0896-6273(01)00338-5 11498052

[B143] Toma H. Winston J. Micci M. A. Shenoy M. Pasricha P. J. (2000). Nerve growth factor expression is up-regulated in the rat model of L-arginine-induced acute pancreatitis. *Gastroenterology* 119 1373–1381. 10.1053/gast.2000.19264 11054396

[B144] Vardanyan M. Melemedjian O. K. Price T. J. Ossipov M. H. Lai J. Roberts E. (2010). Reversal of pancreatitis-induced pain by an orally available, small molecule interleukin-6 receptor antagonist. *Pain* 151 257–265. 10.1016/j.pain.2010.05.022 20599324 PMC3313485

[B145] Vera-Portocarrero L. Westlund K. N. (2005). Role of neurogenic inflammation in pancreatitis and pancreatic pain. *Neurosignals* 14 158–165. 16215298 10.1159/000087654 PMC2766588

[B146] Vera-Portocarrero L. P. Ossipov M. H. King T. Porreca F. (2008). Reversal of inflammatory and noninflammatory visceral pain by central or peripheral actions of sumatriptan. *Gastroenterology* 135 1369–1378. 18694754 10.1053/j.gastro.2008.06.085 PMC4028637

[B147] Vera-Portocarrero L. P. Westlund K. N. (2004). Attenuation of nociception in a model of acute pancreatitis by an NK-1 antagonist. *Pharmacol. Biochem. Behav.* 77 631–640. 10.1016/j.pbb.2004.01.004 15006476

[B148] Vigna S. R. Shahid R. A. Liddle R. A. (2014). Ethanol contributes to neurogenic pancreatitis by activation of TRPV1. *FASEB J.* 28 891–896. 10.1096/fj.13-236208 24221085 PMC3898649

[B149] Vigna S. R. Shahid R. A. Nathan J. D. Mcvey D. C. Liddle R. A. (2011). Leukotriene B4 mediates inflammation via TRPV1 in duct obstruction-induced pancreatitis in rats. *Pancreas* 40 708–714. 10.1097/MPA.0b013e318214c8df 21602738 PMC3116062

[B150] Wan M. H. Huang W. Latawiec D. Jiang K. Booth D. M. Elliott V. (2012). Review of experimental animal models of biliary acute pancreatitis and recent advances in basic research. *HPB* 14 73–81. 10.1111/j.1477-2574.2011.00408.x 22221567 PMC3277048

[B151] Wang Y. Chen M. (2017). Fentanyl ameliorates severe acute pancreatitis-induced myocardial injury in rats by regulating NF-κB signaling pathway. *Med. Sci. Monit.* 23 3276–3283. 28680032 10.12659/MSM.902245 PMC5510983

[B152] Warzecha Z. Dembiński A. Ceranowicz P. Konturek P. C. Stachura J. Konturek S. J. (1997). Protective effect of calcitonin gene-related peptide against caerulein-induced pancreatitis in rats. *J. Physiol. Pharmacol.* 48 775–787. 9444624

[B153] Warzecha Z. Dembiński A. Ceranowicz P. Konturek P. C. Stachura J. Tomaszewska R. (1999). Calcitonin gene-related peptide can attenuate or augment pancreatic damage in caerulein-induced pancreatitis in rats. *J. Physiol. Pharmacol.* 50 49–62. 10210154

[B154] Warzecha Z. Dembiński A. Ceranowicz P. Stachura J. Tomaszewska R. Konturek S. J. (2001). Effect of sensory nerves and CGRP on the development of caerulein-induced pancreatitis and pancreatic recovery. *J. Physiol. Pharmacol.* 52 679–704. 11787767

[B155] Wick E. C. Hoge S. G. Grahn S. W. Kim E. Divino L. A. Grady E. F. (2006a). Transient receptor potential vanilloid 1, calcitonin gene-related peptide, and substance P mediate nociception in acute pancreatitis. *Am. J. Physiol. Gastrointest. Liver Physiol.* 290 G959–G969. 10.1152/ajpgi.00154.2005 16399878

[B156] Wick E. C. Pikios S. Grady E. F. Kirkwood K. S. (2006b). Calcitonin gene-related peptide partially mediates nociception in acute experimental pancreatitis. *Surgery* 139 197–201. 10.1016/j.surg.2005.08.024 16455328

[B157] Willis W. D. Jr. (1999). Dorsal root potentials and dorsal root reflexes: a double-edged sword. *Exp. Brain Res.* 124 395–421. 10.1007/s002210050637 10090653

[B158] Winston J. H. Toma H. Shenoy M. He Z. J. Zou L. Xiao S. Y. (2003). Acute pancreatitis results in referred mechanical hypersensitivity and neuropeptide up-regulation that can be suppressed by the protein kinase inhibitor k252a. *J. Pain* 4 329–337. 10.1016/s1526-5900(03)00636-9 14622690

[B159] Won M. H. Park H. S. Jeong Y. G. Park H. J. (1998). Afferent innervation of the rat pancreas: retrograde tracing and immunohistochemistry in the dorsal root ganglia. *Pancreas* 16 80–87. 9436867 10.1097/00006676-199801000-00013

[B160] Xia K. K. Shen J. X. Huang Z. B. Song H. M. Gao M. Chen D. J. (2019). Heterogeneity of cannabinoid ligand-induced modulations in intracellular Ca(2+) signals of mouse pancreatic acinar cells in vitro. *Acta Pharmacol. Sin.* 40 410–417. 10.1038/s41401-018-0074-y 30202013 PMC6460482

[B161] Yan L. Li Q. F. Rong Y. T. Chen Y. H. Huang Z. H. Wang Z. Z. (2018). The protective effects of rutaecarpine on acute pancreatitis. *Oncol. Lett.* 15 3121–3126. 29435045 10.3892/ol.2017.7659 PMC5778859

[B162] Yang X. Yao L. Fu X. Mukherjee R. Xia Q. Jakubowska M. A. (2020). Experimental Acute Pancreatitis Models: History. Current Status, and Role in Translational Research. *Front Physiol* 11:614591. 10.3389/fphys.2020.614591 33424638 PMC7786374

[B163] Yekkirala A. S. Roberson D. P. Bean B. P. Woolf C. J. (2017). Breaking barriers to novel analgesic drug development. *Nat. Rev. Drug Discov.* 16 545–564. 28596533 10.1038/nrd.2017.87 PMC5675565

[B164] Zaydman M. A. Silva J. R. Cui J. (2012). Ion channel associated diseases: overview of molecular mechanisms. *Chem. Rev.* 112 6319–6333. 23151230 10.1021/cr300360k PMC3586387

[B165] Zheng J. (2013). Molecular mechanism of TRP channels. *Compr. Physiol.* 3 221–242. 23720286 10.1002/cphy.c120001 PMC3775668

[B166] Zyromski N. J. Mathur A. Pitt H. A. Wade T. E. Wang S. Swartz-Basile D. A. (2009). Cannabinoid receptor-1 blockade attenuates acute pancreatitis in obesity by an adiponectin mediated mechanism. *J. Gastrointest. Surg.* 13 831–838. 10.1007/s11605-009-0824-8 19225848

