# Supplementary material for: Molecular mechanisms of pain in acute pancreatitis: recent basic research advances and therapeutic implications
Source: Front Mol Neurosci. 2023 Dec 22;16:1331438. doi: 10.3389/fnmol.2023.1331438 (PMC10771850; doi:10.3389/fnmol.2023.1331438)
Supplement: Supplementary file 1 [file Table_1.docx]

**Supplementary Table 1 |** *In vitro* studies on SP/NK1R in freshly isolated PACs.

| **Pancreas stimuli** | **Mechanism** | **Intervention** | **Effects** | **Ref** |
| --- | --- | --- | --- | --- |
| CER (10^-12^ to 10^-6^ M for 45 min);  SP (1 μM for 45 min, 37°C) | SP/NK1R-NF-κB- Chemokines;  CER-NF-κB- Chemokines | NBD (selective NF-κB inhibitor), 50 μM for 2 h | NF-κB inhibitor inhibited SP- or CER-induced chemokine production (MCP-1, MIP-1α, and MIP-2) | Ramnath *et al*., 2006(Ramnath and Bhatia, 2006) |
| CER (10^–7^ M for 30 and 60 min);  NaHS (H_2_S donor drug, 10, 50, and 100 μM for 30 min) | CER-H_2_S-SP/NK1R-proinflammation | PAG (a CSE (H_2_S synthase) inhibitor), 2, 3, and 4 mM for 30 and 60 min  . | 1) PAG blocked CER-induced SP, *Ppt-a,* and *Nk1r* upregulation;  2) NaHS increased SP production and up-regulated expressions of *Ppt-a* and *Nk1r* | Tamizhselvi *et al*., 2007(Tamizhselvi et al., 2007) |
| SP (1 μM for 0, 3, 5, 10, 15, 30, and 45 min, 37°C) | SP/NK1R-SFK-MAPKs-NF-κB/AP-1/STAT3-Chemokines | PP2 (SFK inhibitor), 1 and 10 μM for 30 min;  CP96345 (NK1R antagonist), 1 μM for 30 min | NK1R or SFK inhibition reduced SP mediated up-regulations of SFK, MAPKs (ERK, JNK), STAT3, NF-κB, AP-1, and chemokine productions (MCP-1, MIP-1α, and MIP-2) | Ramnath *et al*., 2009(Ramnath et al., 2009) |
| CER (10^-12^ to 10^-7^ M for 60 min, 37°C) | CER-MAPKs-NF-κB/AP-1- SP/NK1R | SP600125 (JNK inhibitor), 10, 30, and 50 μM;  PD98059 (MEK inhibitor), 10, 30, and 50 μM;  Bay 11-7082 (NF-κB inhibitor), 10, 30, and 50 μM;  Devazepide (CCK1 antagonist), 100 pM to 10 μM for 30 min before addition of CER (10^-7^ M) | CCK1, MAPKs, or NF-κB inhibition blocked CER-induced SP/NK1R activation | Koh *et al*., 2010(Koh et al., 2010) |
| CER (10^-7^ M for 2, 5, 15, 30, and 60 min, 37°C) | CER-PKC-MAPKs- NF-κB-SP/NK1R | Gö6976 (PKC-α inhibitor), 1, 5, and 10 nM before CER for 45 min;  Rottlerin (PKC-δ inhibitor), 1, 5, and 10 μM before CER for 45 min. | PKC inhibition reversed CER-induced upregulations of MAPKs (ERK1/2 and JNK) and SP/NK1R | Koh *et al*., 2011(Koh et al., 2011a) |
| CER (10^-7^ M for 0-120 min) | SP and NEP | / | CER deceased NEP activity and mRNA expression | Koh *et al*., 2011(Koh et al., 2011b) |
| CER (10^-7^ M for 30, 60, 90, and 120 min, 37°C);  SP (10 ^-6^ M for 30, 60, 90, and 120 min, 37°C);  GR73,632 (NK1R agonist), (10^-6^ M-10^-10^ M for 60 min, 37°C) | CER-PKC-MAPKs- NF-κB-SP/NK1R and SP/NK1R- PKC-MAPKs- NF-κB | Gö6976 (PKC-α/β inhibitor), 10 nM;  Rottlerin (PKC-δ inhibitor), 5 μM;  PD98059 (MEK inhibitor), 30 μM;  SP600125 (JNK inhibitor), 30 μM;  Bay11-7082 (NF-κB inhibitor), 30 μM.  CP96345 (NK1R antagonist), 1μM | 1) NK1R antagonism abolished SP-induced but not CER-induced up-regulated expressions of *Ppt-a* and *Nk1r* mRNA;  2) NK1R agonist up-regulated SP levels while SP increased NK1R expression;  3) PKC, MAPKs, or NF-κB inhibition reduced SP induced *Ppt-a* and *Nk1r* up-regulation as well as NK1R agonist induced SP production | Koh *et al*., 2012(Koh et al., 2012) |
| SP (1 μM for 0, 5, 15, 30, 60, and 120 min) | SP-PKC-MAPKs-LTB_4_ | / | SP induced LTB_4_ production *via* PKC-α/MAPK signalling pathway | Li *et al*., 2018(Li et al., 2018) |
| SP (5 μM for 30 min) | SP/NK1R- NF-κB | CQCQD, 12.5, 25, and 50 μM, 30 min;  CP96345 (NK1R antagonist), 1 μM for 30 min;  Baicalin, emodin, and magnolol, 12.5, 25, and 50 μM for 30 min | CQCQD or its main ingredient (baicalin, emodin, and magnolol) suppressed SP-induced NK1R internalisation and upregulations of β-arrestin1, NF-κB p65, and p-IκB | Han *et al*., 2021(Han et al., 2021) |

*SP,* *substance P; NK1R,* *neurokinin 1-receptor; PACs, pancreatic acinar cells; CER, caerulein; NF-κB,* *nuclear factor kappa-B; MCP-1,* *monocyte chemoattractant protein-1; MIP-1α,* *macrophage inflammatory protein-1α; MIP-2,* *macrophage inflammatory protein-2; NaHS, sodium hydrosulphide; H_2_S,* *hydrogen sulphide; CSE,* *cystathionine-γ-lyase; PPT-A, preprotachykinin-A gene; MAPK,* *mitogen-activated protein kinase; AP-1, activator protein-1; SFK,* *Src family kinase; ERK, extracellular signal-regulated kinase; JNK, c-Jun N-terminal kinase; STAT3,* *signal transducer and activator of transcription 3; MEK, MAPK/ERK kinase; CCK, cholecystokinin; PKC, protein kinase C; NEP, neutral endopeptidase; LTB_4_, leukotriene B_4_; CQCQD, chaiqin chengqi decoction; I-κB,* *inhibitor of NF-κB*

**Supplementary Table 2 |** *In vivo* studies on SP/NK1R in experimental AP models.

| **AP model** | **Species** | **Pain assessment** | **Mechanism** | **Intervention** | **Effects** | **Ref** |
| --- | --- | --- | --- | --- | --- | --- |
| CER (12 × 50 μg/kg/h, i.p.) | Mice | NR | SP and NK1R | NK1R knockout | Genetic NK1R depletion alleviated pancreatic injury and associated lung injury in CER-AP | Bhatia *et al*., 1998(Bhatia et al., 1998) |
| CDE diet (up to 10 d) | Mice | NR | SP, NK1R and NEP | NK1R; NEP knockout | Genetic NK1R depletion reduced pancreatic injury and improved survival in CDE-AP, while NEP depletion showed opposite effects | Maa *et al*., 2000(Maa et al., 2000) |
| CER (6-12 × 50/100 μg/kg/h, i.p.)  CER (10 μg/kg/h, i.v. via femoral vein, for 2 h) | Mice (CER, i.p.)  Rat (CER, i.v.) | NR | SP and NK1R | *In mice*: NK1R knockout;  SR 14333 (NK1R antagonist), 1 mg/kg, i.p.;  HOE 140 (B2-R antagonist), 0.1 nM/kg, i.v.;  *In rats*: [Sar^9^Met(O_2_)^11^]SP (NK1R agonist), 4 nM/kg, i.v.;  [βAla^8^]NKA (NK2R agonist), 4 nM/kg, i.v.; [MePhe7]NKB (NK1R agonist), 4 nM/kg, i.v.;  RP 67580, CP 96345 (NK1R antagonist), 1 μM/kg/h, i.v. | 1) NK1R agonist but not NK2R, NK3R agonist induced pancreatic plasma extravasation in CER-AP mice and rats;  2) NK1R genetic depletion/antagonism alleviated pancreatic extravasation, hyperamylasemia, and pancreatic and lung MPO activity in CER-AP mice | Grady *et al*., 2000(Grady et al., 2000) |
| ERCP (30% meglumine, i.d.) | Rat | NR | NK1R | CP 96345 (NK1R antagonist), 1.0 μmol/kg, i.d., with 30% meglumine | NK1R antagonism reduced NK1R internalisation, pancreatic oedema, MPO activity, and histological score of ERCP-induced AP. | He *et al*., 2003(He et al., 2003) |
| NaTC (30 g/L, 0.1 ml/min/kg, i.d.) | Rat | NR | NK1R/NK2R and intestinal mucosal injury | / | Overexpression of NK1R in distal ileum was associated with increased intestinal permeability and mucosal pathological score in NaTC-AP | Shi *et al*., 2003(Shi et al., 2003) |
| DBTC (8 mg/kg, i.v. via tail vein) | Rat | Abdomen von Frey test^*^ and thermal sensitivity^#^, 30 min after intervention | NK1R | CP 99,994 (NK1R antagonist), 1, 5, 10 and 30 mg/kg, i.p. or 1, 5, 10, 15, 20 μg i.t.;  CP 100,263 (the inactive enantiomer), 1, 5, 10, and 30 mg/kg, i.p., or 1, 5, 10, 15, 20 μg i.t. | 1) NK1R protein expression increased significantly in the pancreas on days 3 and 7 after DBTC-AP;  2) NK1R antagonism attenuated nociceptive behavior when applied intraperitoneally but not intrathecally in DBTC-AP | Vera-Portocarrero *et al*., 2004(Vera-Portocarrero and Westlund, 2004) |
| CER (10 × 50 μg/kg/h, i.p.) | Mice | NR | SP and NK1R | CP 96345 (NK1R antagonist), 2.5 mg/kg, i.p., 30 min before or 1 h after the 1^st^ CER injection | 1) There was a time-dependent increase of SP level in pancreas, plasma, and lung in CER-AP;  2) Both prophylactical and therapeutical NK1R antagonism reduced amylase, MPO, and improved histological damage of pancreas and lung in CER-AP | Lau *et al*., 2005(Lau et al., 2005) |
| CER (10 × 50 μg/kg/h, i.p.) | Mice | NR | NK1R and NK2R | CP 96345 (NK1R antagonist), 2.5 mg/kg, i.p., 30 min before or 1 h after the 1^st^ CER | 1) NK1R antagonism reduced both pancreatic and pulmonary SP levels, *Ppt-a* and *Ppt-c* expressions;  2) NK1R antagonism reduced NK1R expression, increased NK2R expression in pancreas while opposite in lung;  3) NK3R was absent in both pancreas and lung | Lau *et al*., 2006(Lau and Bhatia, 2006) |
| CER (50 μg/kg, for 3, 6 and 10 h, i.p.) | Mice | NR | SP/NK1R and chemokine | CP 96345 (NK1R antagonist), 2.5 mg/kg, i.p., 0.5 h before or 1 h after the 1^st^ CER | Blockade of NK1R attenuated chemokine production (MCP-1, MIP-1α, and MIP-2), pancreas and lung injury in CER-AP | Sun *et al*., 2007(Sun and Bhatia, 2007) |
| CER (10 × 50 μg/kg/h, i.p.) | Mice | NR | H_2_S-SP/NK1R-inflammation | PAG (a CSE (H_2_S synthase) inhibitor) 100 mg/kg, i.p., either 1 h before or 1 h after the 1^st^ CER | H_2_S synthase inhibition reduced plasma and pancreatic H_2_S and SP levels, *Ppt-a* and *Ppt-c* mRNA expression in pancreas and lung in CER-AP | Bhatia *et al*., 2008(Bhatia et al., 2008) |
| sPLA2 (300 μg/kg, i.d. final volume of 0.3 mL) | Rat | NR | sPLA2-SP/NK1R and bradykinin/B2 receptor-inflammation | SR 140333 (NK1R antagonist), 120 nmol/kg both i.v. 15 min before and s.c. after AP;  Icatibant (Bradykinin B_2_ receptor antagonist), 100 nmol/kg, s.c., 30 min before and 1 h after AP | NK1R or bradykinin B_2_ receptor antagonism reduced plasma extravasation and TNF-α levels, pancreatic and lung MPO in sPLA2 induced AP | Camargo *et al*., 2008(Camargo et al., 2008) |
| CER (10 × 50 μg/kg/h, i.p.) | Mice | NR | SP/NK1R-SFKs-MAPKs- NF-κB/AP-1-Chemokines | PP2 (selective SFK inhibitor), 0.5, 1.0, and 1.5 mg/kg, i.p., 1 h before or 1 h after the 1^st^ CER;  CP 96345 (NK1R antagonist), 2.5 mg/kg, i.p., 1 h before or 1 h after the 1^st^ CER | 1) SFK blockade alleviated serum amylase, pancreatic MPO, chemokine production, and pancreatic histology in CER-AP;  2) Either NK1R or SFK inhibition reduced pancreatic MAPKs, STAT3, NF-κB, and AP-1 expressions in CER-AP | Ramnath *et al*., 2009(Ramnath et al., 2009) |
| CER (7 × 50 μg/kg/h, i.p.) | Mice | NR | SP/NK1R and galanin | Galantide (the galanin antagonist), 66 μg/kg, i.p., with the first CER or 1 h after the first CER;  L 703,606 (NK1R antagonist), 8 mg/kg, i.p., 30 min before or 2 h after the 1^st^ CER;  Galantide + L 703,606 | Prophylactic or therapeutic treatment with NK1R or galanin antagonist alone, and in combination, ameliorate CER-AP, while combined antagonists does not offer any further benefit to galantide alone | Barreto *et al*., 2010(Barreto et al., 2010) |
| CER (50 μg/kg/h for 3, 6, 10 times, i.p.) | Mice | NR | SP and NEP | NEP, 1 mg/kg, i.v., during 2^nd^ CER injection;  Phosphoramidon (NEP inhibitor), 5 mg/kg, i.v., 1 h before the 6^th^ CER injection;  Thiorphan (NEP inhibitor), 10 mg/kg, i.v., 1 h before the 6^th^ CER injection | 1) NEP mRNA expression was decreased in pancreas and lung in CER-AP;  2) NEP inhibition raised SP levels and exacerbated inflammatory conditions in CER-AP;  3) Exogenous NEP decreased SP levels and attenuated CER-AP | Koh *et al*., 2011(Koh et al., 2011b) |
| CER (7 × 50 μg/kg/h, i.p.) | Mice | NR | SP and NK1R | Maropitant citrate (NK1R antagonist), 8 mg/kg, s.c., after the 1^st^ injection of CER | NK1R antagonism decreased pancreatic SP mRNA expression, plasma amylase, and IL-6 levels, and pancreatic MPO activity in CER-AP | Tsukamoto *et al*., 2018(Tsukamoto et al., 2018) |
| CER (10 × 100 μg/kg/h, i.p.) plus LPS (5 mg/kg, i.p.) after the last CER injection; L-Arg (8%, 2 × 4 g/kg, pH = 7.0, i.p.) | Mice | NR | SP/NK1R-PKCα/MAPK-LTB4/BLT1-AP/ALI | LY 293111 (BLT1 antagonist), 5 mg/kg, i.p., 0.5 h before the 1^st^ injection of CER or L-Arg);  CP 96345 (NK1R antagonist), 5 mg/kg, i.p., 0.5 h before the 1^st^ L-Arg injection | 1) The levels of LTB4 and its specific receptor BLT1 were upregulated in both AP models;  2) BLT blockade attenuated severity of AP and ALI;  3) NK1R antagonism attenuated AP severity and reduced LTB4 levels | Li *et al*., 2018(Li et al., 2018) |
| CER (6 × 50 μg/kg/h, i.p.) | Mice | NR | SP/NK1R and CSE/H_2_S-NF-κB-AP/ALI | Menadione, 10 mg/kg, i.p., after the 1^st^ CER injection | Menadione attenuated the severity of AP and associated ALI by the serum amylase and H_2_S, alleviated pancreatic MPO activity and histopathology score accompanied with reduced expressions of pancreatic *IL-1β*, *CSE*, *PPT-A*, and *NK1R* as well as NF-кB activation | Amiti *et al*., 2019(Amiti et al., 2019) |
| CER (6 × 50 μg/kg/h, i.p.) | Mice | NR | SP/NK1R and CSE/H_2_S- NF-κB- AP/ALI | DADS (diallyl disulfide), 100, 150, 200 μg/kg, i.p., after the 1^st^ CER injection | DADS attenuated the severity of AP and associated ALI by inhibiting histological damage, MPO, serum amylase, TNF-α, H_2_S, CSE, *PPTA*, *NK1R* expression, reduced I-κB degradation and NF-κB translocation in pancreas and lung | Mathan Kumar *et al*., 2020(Mathan Kumar and Tamizhselvi, 2020) |
| CER (7 × 50 μg/kg/h, i.p.) | Mice | Hindpaw von Frey test^&^, 6.5, 7.5, 8.5, and 9 h after the last injection of CER | SP and NK1R | CQCQD, 10 g/kg, 200 μl, gavaged together with the 3^rd^, 5^th^, and 7^th^ CER injection;  CP 96345 (NK1R antagonist), 5 mg/kg, i.p., 30 min before the 1^st^ CER injection | CQCQD ameliorated CER-AP and its associated pain via inhibiting neuron activation-mediated acinar cell SP/NK1R pathways | Han *et al*., 2021(Han et al., 2021) |

^*^Rats were placed individually in Plexiglas cubicle on an elevated, fine fiberglass screen mesh. The von Frey filament was applied to stimulate the abdominal area in 10 applications per trial, with a 10-second interval to allow the rat to cease any response and return to a relatively inactive position. The mean occurrence of withdrawal events in each trial was expressed as the number of responses per 10 applications. Each rat underwent three trials, and the average withdrawal events were calculated. ^#^Rats were placed in Plexiglas cubicle on an elevated glass plate through which a high-intensity light beam shining through. A radiant heat stimulus was applied to the abdominal area, and the light and timer were immediately stopped when the animal withdrew. Withdrawal was defined as either abdominal musculature contraction or lifting the abdomen through postural adjustment, accompanied by head turning toward the stimuli and licking of the abdominal area. ^&^Mice were individually housed in an elevated transparent cage with a wire mesh floor. The Von Frey filament was used to assessed the mechanical paw withdrawal, where rapid paw withdrawal or flinching was considered a positive response. *SP, substance P; NK1R, neurokinin 1-receptor; AP, acute pancreatitis; CER, caerulein; i.p., intraperitoneal; NR, not reported; CDE, choline-deficient and ethionine supplemented; NEP, neutral endopeptidase; i.v., intravenous; B2-R, bradykinin-2 receptor; MPO, myeloperoxidase; ERCP, endoscopic retrograde cholangiopancreatography; i.d., intrapancreatic duct; NaTC, sodium taurocholate; DBTC, dibutyltin dichloride; i.t., intrathecal; DBTC-AP, DBTC-induced acute pancreatitis;* *TRPV1, transient receptor potential vanilloid 1; Ppt-a, preprotachykinin-A gene; Ppt-c, preprotachykinin-C gene; NK2R, neurokinin 2-receptor; NK3R, neurokinin 3-receptor; RTX, resiniferatoxin; MCP-1, monocyte chemoattractant protein-1; MIP-1α, macrophage inflammatory protein-1α; MIP-2, macrophage inflammatory protein-2; CSE, cystathionine-γ-lyase; H_2_S, hydrogen sulphide; sPLA2, secretory phospholipases A2; s.c., subcutaneous; TNF-α, tumour necrosis factor-alpha; SFK, Src family kinase; MAPK, mitogen-activated protein kinase; STAT3, signal transducer and activator of transcription 3; NF-κB, nuclear factor kappa-B; AP-1, activator protein-1; IL-6, interleukin-6; LPS, lipopolysaccharide; L-Arg, L-arginine; PKC-α, protein kinase C-alpha; LTB_4_, leukotriene B_4_; BLT1, leukotriene B4 receptor 1; ALI, acute lung injury; IL-1β, interleukin-1β; I-κB, inhibitor of NF-κB; CQCQD, chaiqin chengqi decoction.*

**Supplementary Table 3 |** Studies on CGRP in experimental AP models.

| **AP model** | **Species** | **Pain assessment** | **Mechanism** | **Intervention** | **Effects** | **Ref** |
| --- | --- | --- | --- | --- | --- | --- |
| CER (10 μg/kg/h, s.c., infusion at rate of 1 mL/h, for 5 h) | Rat | NR | CGRP and sensory nerves | CGRP, 10 μg/kg, s.c., 30 min before CER and 3 h later;  Capsaicin (neurotoxic dose), s.c., 100 mg/kg | CGRP treatment but not capsaicin attenuated the severity of CER-AP by reducing pancreatic weight, protein content, improving pancreatic blood flow, synthesis of RNA content and DNA, and histological changes | Warzecha *et al*., 1997(Warzecha et al., 1997) |
| CER (10 μg/kg/h, s.c., infusion at rate of 1 mL/h, for 5 h) | Rat | NR | CGRP | CGRP, 10 μg/kg, s.c., 30 min before the 1^st^ CER infusion and 3 h later;  CGRP, 10 μg/kg, 1, 4, and 7 h after the last CER infusion | CGRP treatment before and during induction protected against CER-AP, while CGRP treatment after CER-AP aggravated pancreatic damage | Warzecha *et al*., 1999(Warzecha et al., 1999) |
| CER (10 μg/kg/h, s.c., infusion at rate of 1 mL/h, for 5 h, once a week, twice in total) | Rat | NR | CGRP and sensory nerves | CGRP, 10 μg/kg, s.c., 3 times daily for 2 weeks;  Capsaicin (stimulatory dose), 0.5 mg/kg, s.c., 3 times daily for 2 weeks | Prolonged activity of sensory nerves and the presence of CGRP during pancreatic regeneration after AP led to pancreatic exocrine insufficiency and delay in pancreatic regeneration | Warzecha *et al*., 2000(Warzecha et al., 2000) |
| CER (10 μg/kg/h, s.c., infusion at rate of 1 mL/h, for 5 h, single or once a week, twice in total) | Rat | NR | CGRP and sensory nerves | Capsaicin, 0.5 mg/kg (stimulatory dose), s.c., 30 min before the 1^st^ CER infusion and 3 h later  Capsaicin, 100 mg/kg (neurotoxic dose), s.c., 6 injections over 3 consecutive days, 10 d before induction of AP;  CGRP, 10 μg/kg, s.c., 30 min before the 1^st^ CER infusion and 3 h later | 1) Stimulation of sensory nerves and administration of CGRP protected pancreatic damage before and during the induction of CER-AP;  2) Persistent activity of sensory nerves and presence of CGRP after CER-AP aggravated pancreatic damage and led to chronic pancreatitis | Warzecha *et al*., 2001(Warzecha et al., 2001) |
| IRI | Rat | NR | CGRP and sensory nerves | Capsaicin (stimulatory dose), 0.5 mg/kg, s.c., 1 h before IRI;  Capsaicin (neurotoxic dose), 100 mg/kg, s.c., 6 injections over 3 consecutive days, 10 d before induction of AP;  CGRP, 10 μg/kg, s.c. | Stimulation of sensory nerves and pretreatment with CGRP attenuated pancreatic damage, while ablation of these nerves aggravated this effect in IRI-AP partially reversed by CGRP prior IRI | Dembiński *et al*., 2003(Dembiński et al., 2003) |
| L-Arg (2 × 2.5 mg/kg/h; i.p.) | Rat | NR | CGRP-nociceptive pathway | CGRP8-37 (CGRP antagonist), 50 μg, i.t., 2 h before L-Arg | Intrathecal CGRP inhibition reduced the activation of nociceptive pathway in ARG-AP | Wick *et al*., 2006(Wick et al., 2006b) |
| GDOC (5 mM; 10 min, 1.2 mL/kg, the common bile duct) and CER (5 μg/kg/h, i.v. over 6 h after induction SNP) | Rat | NR | CGRP | CGRP, 10 μg/kg, infusion 6 h, at the initiation or 1 h after AP induction | 1) Exogenous CGRP induced activation of adenylate cyclase resulting in reduced NF-κB activation and inflammatory cytokines and ICAM-1;  2) Both prophylactic and therapeutic application of CGRP reduced the pancreatic morphologic damage of the SNP model | Schneider *et al*., 2009(Schneider et al., 2009) |
| CER (8 × 0.2 μg/injection/h, i.p.) | Mice | NR | CGRP receptor RAMP1 | RAMP1 knockout | RAMP1 knockout worsened CER-AP with significantly increased serum lipase, pancreatic edema, apoptosis, and immune-cell infiltration | Jochheim *et al*., 2019(Jochheim et al., 2019) |
| *In vivo:* CER (7 × 50μg/kg/h; i.p.) and LPS (1 × 10 mg/kg, at the same time as the fifth CER injection);  *In vitro:* CER (10^-8^ M; 4 h) | Mice;  AR42J cells | NR | CGRP-MAPK- NF-κB/STAT3- Cytokines | *In vivo:* CGRP knockout; RUT (an alkaloid component of  *Evodia rutaecarpa*), 25/50/100 mg/kg/day, i.g., 24 h after the 1^st^ CER injection  *In vitro:* CGRP-overexpression; CGRP-shRNA; RUT, 70 μg/mL, 6 h after CER treatment | 1) RUT ameliorated pathological damage as well as serum IL-6, TNF-α and upregulated IL-10 partly through the suppression of MAPK and NF-κB via CGRP in CER/LPS-induced AP;  2) RUT inhibited the activation of MAPK, NF-κB and STAT3 signaling pathway via CGRP in AR42J AP model cells | Huang *et al.,*  2021(Huang et al., 2021) |

*CGRP, calcitonin gene-related peptide; AP, acute pancreatitis; CER, caerulein; s.c., subcutaneous; NR, not reported; CER-AP, caerulein-induced acute pancreatitis; IRI, ischaemia-reperfusion injury; IRI-AP, ischaemia-reperfusion injury-induced acute pancreatitis; L-Arg, L-arginine; i.p., intraperitoneal; i.t., intrathecal; ARG-AP, L-arginine-induced acute pancreatitis; GDOC,* *glycodeoxycholic acid; i.v., intravenous; SNP, severe necrotizing pancreatitis; NF-κB, nuclear factor kappa-B; ICAM-1, intercellular adhesion molecule-1; RAMP1, receptor-activity-modifying-protein-1; LPS, lipopolysaccharide; i.g., intragastrically; MAPK, mitogen-activated protein kinase; STAT3, signal transducer and activator of transcription 3; RUT,* *rutaecarpine; IL-6, interleukin-6; TNF-α, tumor necrosis factor α.*

**Supplementary Table 4 |** Studies on TRPV1 in experimental AP models.

| **AP model** | **Species** | **Pain assessment** | **Mechanism** | **Interventions** | **Effects** | **Ref** |
| --- | --- | --- | --- | --- | --- | --- |
| CER (12 × 50 μg/kg/h, i.p.) | Mice | NR | TRPV1-SP/NK1R- Neuroinflammation | Capsazepine (TRPV1 antagonist), 100 μmol/kg, s.c., 4 injections at 4-h intervals, started 1 h before the 1^st^ CER injection | TRPV1 inhibition reduced SP release, NK1R internalisation, and alleviated CER-AP | Nathan  *et al*., 2001(Nathan et al., 2001) |
| CER (10 μg/kg/h, i.v. via tail vein) | Rat | NR | TRPV1- SP/NK1R- Neuroinflammation | Capsaicin (TRPV1 agonist), 1 or 2 μM/kg, i.v.;  Capsazepine (TRPV1 antagonist), 1.8 mg/kg, i.v.;  CP 96345 (NK1R antagonist), 1 mg/kg, i.v. | 1) TRPV1 activation induced dose-dependent plasma extravasation in the pancreas reversed by TRPV1 or NK1R antagonism;  2) NK1R antagonism alleviated pancreatic plasma extravasation, MPO, and histological inflammation in CER-AP | Hutter *et al*., 2005(Hutter et al., 2005) |
| CER (6 × 50 μg/kg/h, i.p.) | Rat | NR | TRPV1- SP/NK1R- Neuroinflammation | RTX (an excitotoxin that  desensitizes TRPV1), 10 μg,  ganglionectomy | Surgical disruption or TRPV1 desensitisation inhibited NK1R internalisation and reduced MPO and histological score of CER-AP | Noble *et al*., 2006(Noble et al., 2006) |
| CER (12 × 50 μg/kg/h, i.p.) | Mice | NR | TRPV1 | TRPV1 knockout;  RTX (an excitotoxin that  desensitises TRPV1), 50 μg/kg, s.c., 48 h before 1^st^ CER injection | Pharmacological disruption of TRPV1 but not TRPV1 knockout protected against CER-AP | Romac  *et al*., 2008(Romac et al., 2008) |
| CER (6 × 50 μg/kg/h, i.p.) | Mice | Abdomen von Frey test^*^, 30 min after last CER injection | Proteinase/PAR2/  TRPV1 cascade | Nafamostat mesylate (proteinase inhibitor), 0.1, 1.0, and 10 mg/kg, i.p., 15 min after the last CER injection;  Capsazepine (TRPV1 antagonist), 15 mg/kg, s.c., 10 min after the last injection of CER or two doses at 30 min before 1^st^ and 4^th^ CER injection;  Trypsin (endogenous PAR2 agonist) or SLIGRL-NH2 (PAR2 activating peptide), 25 μl, i.d., 2 h before sampling | 1) Post treatment with nafamostat mesylate or capsazepine abolished referred allodynia/hyperalgesia in CER-AP, but did not impact pancreatic injury;  2) Preventive treatment with capsazepine had no effect on pain or pancreatic damage in CER-AP;  3) Pancreatic PAR2 activation increased spinal c-Fos expression, blocked by proteinase or TRPV1 inhibition | Nishimura  *et al*., 2010(Nishimura et al., 2010) |
| CER (6 × 50 μg/kg/h， i.p.) | Mice | Abdomen von Frey test^*^ 20-30 min after tacrolimus administration | Calcineurin and TRPV1 | Tacrolimus (calcineurin blocker), 10 mg/kg, i.p., 10 min and 24 h after the last CER injection;  NNC 55-0396 (T-type Ca^2+^ channel blocker), 10 mg/kg, i.p., 30 min before tacrolimus treatment;  SB366791 (TRPV1 blocker), 0.5 mg/kg, i.p., 30 min before tacrolimus treatment | 1) Tacrolimus had no effect on referred hyperalgesia in acute phase, but caused relapse of referred hyperalgesia in recovery phase of CER-AP;  2) TRPV1 blocker but not T-type Ca^2+^ blocker abolished tacrolimus-induced relapse of referred hyperalgesia in the recovery phase of CER-AP | Terada  *et al*., 2017(Terada et al., 2017) |
| L-Arg (2 × 2.5 g/kg, i.p.) | Rat | Abdominal contractions^#^, 12, 24, and 36 h after AP induction | TRPV1, CGRP, and SP | Capsaicin (TRPV1 agonist), 1 mg/kg, i.d.;  Capsazepine (TRPV1 antagonist), 25 μg/kg, s.c., 1 h before AP induction and re-treat every 12 h;  CGRP_8–37_ (CLR antagonist), 50 μg, i.t., 22 h after AP induction;  SR140333 (NK1R antagonist), 30 μg, i.t., 22 h after AP induction | 1) Pancreatic sensory nerves co-expressed TRPV1, SP, and CGRP;  2) Necrotising AP activated TRPV1 on pancreatic sensory nerves to release CGRP and SP in the dorsal horn to mediate nociception;  3) Systemic antagonism of TRPV1 and central antagonism of SP and CGRP receptors inhibited nociception in ARG-AP | Wick  *et al*., 2006(Wick et al., 2006a) |
| *In vivo:*  NaTC (2%, i.d.);  CER (6 × 50 μg/kg/h, i.p.)  *In vitro:* TLCS (500 μM) | Mice;  PACs | NR | NaTC-5-LO-LTB_4_-TRPV1-SP-Neuroinflammation | TRPV1 knockout;  RTX (an excitotoxin that  desensitizes TRPV1), 14 μg/mL, 5 μL/min, i.d., 10 min;  MK886 (a drug that inhibits LTB_4_  biosynthesis), 10 mg/kg, i.p., 1 h before surgery | 1) Desensitisation of TRPV1 resulted in significant inhibition of NaTC-AP;  2) TRPV1 knockout significantly inhibited pancreatic histopathology and MPO levels but had no effect on pancreatic oedema or serum amylase in NaTC-AP;  3) LTB_4_ inhibition reduced pancreatic histopathology, MPO, and expression of 5-LO (IHC) in NaTC-AP | Shahid  *et al*., 2015(Shahid et al., 2015) |
| NaTC (5%, i.d.) | Rat | NR | MiR-21-3p-TRP channels-Apoptosis | MiR-21-3p mimics, 12 h before modelling, i.v. via tail vein;  MiR-21-3p inhibitors, 12 h before modelling, i.v. via tail vein;  Gd3^+^ (TRP channel blocker), 12 h before modelling, i.v. via tail vein | 1) MiR-21-3p expression was increased in pancreas and lung in NaTC-AP;  2) Up-regulation of miR-21-3p aggravated pancreatic and lung injury by activating TRP signalling pathway in NaTC-AP;  3) Down-regulation of miR-21-3p or TRP inhibition promoted apoptosis of PACs and alleviated NaTC-AP | Wang  *et al*., 2018(Wang et al., 2018) |
| NaTC (5%, 1.0mL/kg, i.d.) | Rat | NR | TRPV1, CGRP and cytokines | Rutaecarpine (an alkaloid component of  *Evodia rutaecarpa*), 30/100/300 μg/kg, 20 min prior to surgery, injected into the sublingual vein;  Capsazepine (TRPV1 antagonist), 3 mg/kg, 30 min prior to surgery, injected into the sublingual vein | Rutaecarpine decreased amylase, IL-6, TNF-α and the severity of pancreatic pathological changes, but increased serum IL-10 concentration, which mediated by the release of CGRP via activation of TRPV1 | Yan *et al.*, 2018(Yan et al., 2018) |
| EtOH (1.32 g/kg, i.p.) + POA (2 mg/kg, i.p., 1 h later) | Mice | NR | FAEE-TRPV1-Neuroinflammation | TRPV1 knockout;  AMG9810 (TRPV1 antagonist), 30 mg/kg, i.p., 30 min before EtOH/POA administration | Pharmacological inhibition or gene depletion of TRPV1 alleviated FAEE-AP | Vigna  *et al*., 2014(Vigna et al., 2014) |
| Radiocontrast solutions, pH = 6.0/6.9/7.3, i.d. | Rat | NR | Low pH contrast solution-TRPV1-AP | RTX (an excitotoxin that  desensitises TRPV1), 10 μg/400 μL added to contrast solution, i.d. | Low pH contrast solutions (6.9 and 6.0) caused AP which was blocked by TRPV1 inhibition, while pH 7.3 did not cause AP | Noble  et al., 2008(Noble et al., 2008) |
| PDL;  LTB_4_, 15 μg/kg by celiac artery injection | Rat | NR | PDL-LTB_4_-TRPV1- Neuroinflammation | Capsazepine (TRPV1 antagonist), 100 μmol/kg, s.c., 30 min before model;  MK-886 (a drug that inhibits LTB_4_  biosynthesis), 10 mg/kg i.p., 1 h before model | 1) TRPV1 antagonism reduced LTB_4_- or PDL-induced pancreatic injury;  2) LTB_4_ inhibition alleviated PDL-AP | Vigna  et al., 2011(Vigna et al., 2011) |

^*^Mice were placed on a raised wire floor covered with a clear plastic box, the upper abdomen of each mouse was stimulated with three different strength levels of Von Frey filaments in ascending order of force for a total of 10 times. Nociceptive behavior was scored as follows: 0, no response; 1, immediate escape or licking/scratching of the site stimulated with filaments; 2, strong retraction of the abdomen or jumping. Data were presented as a total score over the 10 stimulations. ^#^Six NiCr wire electrodes were implanted bilaterally in the upper half of the external oblique muscle of the abdominal wall to amplify electrical activity of the abdominal muscles and record resposnes. *TRPV1, transient receptor potential vanilloid 1; AP, acute pancreatitis; CER, caerulein; NR, not reported; SP, substance P; NK1R, neurokinin 1-receptor; s.c., subcutaneous; CER-AP, caerulein-induced acute pancreatitis; i.p., intraperitoneal; RTX, resiniferatoxin;* *PAR2, proteinase-activated receptor-2; i.d., intrapancreatic duct; L-Arg, L-arginine; CGRP, calcitonin gene-related peptide; i.t., intrathecal;* *CLR, calcitonin-like receptor; ARG-AP, L-arginine-induced acute pancreatitis; NaTC, sodium taurocholate; TLCS, taurolithocholic acid 3-sulphate disodium salt; PACs, pancreatic acinar cells; MPO, myeloperoxidase; 5-LO, 5-lipoxygenase; IHC, immunohistochemistry; LTB_4_, Leukotriene B_4_; MiR, microRNA; TRP, transient receptor potential channels; i.v., intravenous; EtOH, ethanol; POA, palmitoleic acid; FAEE-AP, fatty acid ethyl ester-induced acute pancreatitis; PDL, pancreatic duct ligation; PDL-AP, pancreatic duct ligation-induced acute pancreatitis.*

**Supplementary Table 5 |** Studies on TRPA1 in experimental AP models.

| **AP model** | **Species** | **Pain assessment** | **Mechanism** | **Interventions** | **Effects** | **Ref** |
| --- | --- | --- | --- | --- | --- | --- |
| CER (8 × 50 μg/kg/h, i.p.) | Mice | Pain-related behavioral test^*^ for 15 min periods at 9, 10, 12, 14, and 24 h after treatment | TRPA1/TRPV1-neuroinflammation | HC-030031 (TRPA1 antagonist), 100 or 300 mg/kg, i.p.;  AMG 9810 (TRPV1 antagonist), 100 and 300 mg/kg, i.p.;  HC-030031 + AMG 9810, 50 or 100 mg/kg, i.p. | TRPV1 and/or TRPA1 antagonism attenuated pain and pancreatic inflammation in a synergistic way in CER-AP | Schwartz *et al*., 2011(Schwartz et al., 2011) |
| CER (8 × 50 μg/kg/h, i.p., twice weekly, for 10 weeks) | Mice | Pain-related behavioral test^*^ before and after 3 weeks antagonist injection later | TRPA1/TRPV1-neuroinflammation | A-967079 (TRPA1 antagonist) and/or A-889425 (TRPV1 antagonist), 1 h before the 1^st^ CER or vehicle injection | Pain-related behaviours during AP to CP transition was accompanied by increased expressions of TRPV1, TRPA1, and pERK in pancreatic afferents, blocked by TRP channel antagonist | Schwartz *et al*., 2013(Schwartz et al., 2013) |
| CER (6 × 50 μg/kg/h, i.p.) | Mice | Abdomen von Frey test^#^ | PAR2-TRPA1/TRPV1-neuroinflammation | AP18 (TRPA1 inhibitor), 10 mg/kg, i.p.;  SB366791 (TRPV1 inhibitor), 0.1 or 0.5 mg/kg, i.p.;  SLIGRL-NH2 (PAR2-acting peptide), 15 nmol/mice, i.d. | 1) TRPA1 or TRPV1 inhibition blocked PAR2-triggered pancreatic nociception;  2) TRPV1 but not TRPA1 inhibition alone or TRPA1 inhibition plus partial TRPV1 inhibition reversed CER-AP referred hyperalgesia | Terada *et al*., 2013(Terada et al., 2013) |
| CER (12 × 50 μg/kg/h, i.p.) | Mice | Abdomen von Frey test^#^, 30 min after the last CER injection | H_2_S-Cav3.2 T type Ca^2+^ channels, TRPA1/TRPV1 channels and AP pain | AP18 (TRPA1 inhibitor), 10 mg/kg, i.p.;  NNC55-0396 (a T-type Ca^2+^ channel blocker), 1, 3, or 10 mg/kg, i.p.;  Zinc chloride (selective Cav3.2 inhibitor), 34 or 340 mg/kg, i.p., or ascorbic acid (selective Cav3.2 inhibitor), 10 or 20 mg/kg, i.p.;  Cav3.2 knockdown | 1) Exogenous H_2_S-induced TRPA1 and Cav3.2 mediated pancreatic nociception in naive mice;  2) Cav3.2 but not TRPA1 inhibition alone or TRPA1 inhibition plus partial Cav3.2 inhibition reversed CER-AP referred hyperalgesia | Terada et al., 2015(Terada et al., 2015) |

^*^Mice were placed in Plexiglas boxes and their exploratory behaviors were monitored using photoelectrical sensors. Photoelectric beams were spaced 1.5 cm apart, providing 0.75 cm spatial resolution. The TruScan software was employed to analyze various parameters, including the time spent in different areas, path information, distance traveled, total movements simultaneously in the X-Y plane, and the duration each mouse spent in the vertical plane or standing position that necessitated a stretch of the abdominal muscles. This position is assumed to be uncomfortable in the context of abdominal hypersensitivity. ^#^Mice were placed on a raised wire floor covered with a clear plastic box, the upper abdomen of each mouse was stimulated with three different strength levels of Von Frey filaments in ascending order of force for a total of 10 times. Nociceptive behavior was scored as follows: 0, no response; 1, immediate escape or licking/scratching of the site stimulated with filaments; 2, strong retraction of the abdomen or jumping. Data were presented as a total score over the 10 stimulations. *TRPA1, transient receptor potential ankyrin 1; AP, acute pancreatitis; CER, caerulein; i.p., intraperitoneal; TRPV1, transient receptor potential vanilloid 1; CER-AP, caerulein-induced acute pancreatitis; CP, chronic pancreatitis; pERK, phosphorylated extracellular signal-regulated kinase; TRP, transient receptor potential; PAR2, proteinase-activated receptor-2; i.d., intrapancreatic duct; H_2_S, hydrogen sulphide.*

**Supplementary Table 6 |** Studies on TRPV4 in experimental AP models.

| **AP model** | **Species** | **Pain assessment** | **Mechanism** | **Intervention** | **Key findings** | **Ref** |
| --- | --- | --- | --- | --- | --- | --- |
| CER (12 × 50 μg/kg/h, i.p.)  4αPDD (TRPV4 agonist), 0.025 mg/mL, i.d.;  MO (TRPA1 agonist), 10 mg/mL, i.d.;  15dPGJ_2_ (endogenous TRPV1 agonist), 600 μM, i.d.;  HNE (endogenous TRPA1 agonist), 100 μM, i.d.; | Mice | Pain-related behavioral test^*^ | TRPV4 and TRPA1 | TRPV4 or TRPA1 knockout | 1) TRPA1 and TRPV4 agonist activated nociceptive spinal neurons, and TRPA1 but not TRPV4 agonist induced pancreatic inflammation  2) Genetic deletion of TRPV4 and TRPA1 attenuated pain during CER-AP, and deletion of TRPA1 but not TRPV4 attenuated pancreatic inflammation | Ceppa *et al*., 2010(Ceppa et al., 2010) |
| CER (6 × 50 μg/kg/h, i.p.) | Mice | Assessment of nocicepsive behavior^#^ | TRPV4 and TRPA1 | Compound 16-8/16-19 (TRPV4/TRPA1 dual-inhibitors), 10 mg/kg, i.p. | TRPV4/TRPA1 inhibition attenuated pain and inflammation in CER-AP | Kanju *et al*., 2016(Kanju et al., 2016) |
| Yoda1 (Piezo1 agonist, 0.4 mg/kg,  partial PDL  and i.d.);  Partial PDL and infused 50/400 μl HEPES (10 mM, pH 7.6) at the rate of 5/80 μl/min for 10/5 min to produce low-/high-pressure | Mice  PACs | NR | TRPV4 and Piezo1 | TRPV4 knockout  Piezo1 knockout | 1) Piezo1 activation caused a prolonged elevation in intracellular Ca^2+^ levels, mitochondrial depolarization, intracellular trypsin activation, and cell death in PACs;  2) Piezo1 stimulation triggered TRPV4 channel opening which was responsible for the sustained elevation in intracellular Ca^2+^ that caused intracellular organelle dysfunction  3) Piezo1 knockout mice were protected from Piezo1 agonist and duct ligation–induced pancreatitis;  4) TRPV4 knockout mice were protected from Piezo1 agonist and duct ligation–induced pancreatitis | Swain *et al*., 2020(Swain et al., 2020) |

^*^Mice were placed individually in the center of an open field, and the number of abdominal retractions, squashes, and licking behaviors were recorded over a 30-minute period. ^#^Mice were housed in individual cages and video-recorded throughout the entire experiment. Linear movement was quantified as one event when mice crossed the median plane of the cage. *TRPV4, transient receptor potential vanilloid 4; AP, acute pancreatitis; CER, caerulein; i.p., intraperitoneal; TRPA1, transient receptor potential ankyrin 1; i.d., intrapancreatic duct; PDL, pancreatic duct ligation; PACs, pancreatic acinar cells; NR, not reported.*

**Supplementary Table 7 |** *In vitro* studies on CBRs in freshly isolated PACs.

| **Pancreas stimuli** | **Species** | **Mechanism** | **Intervention** | **Effects** | **Ref** |
| --- | --- | --- | --- | --- | --- |
| CER (10^-9^ M)  CCh (10^-5^ M)  KCl (75 mM) | Guinea pig and rat PACs | CB1R, CB2R; exocrine pancreatic secretion | WIN 55,212 (nonselective CBR agonist),  10^-6^ M;  AM630 (CB2R antagonist), 10^-7^ M;  AM251 (CB1R antagonist), 10^-7^ M | CBR agonist inhibited KCl-stimulated amylase secretion, reduced by CBR antagonists;  CBR agonist did not affect basal or CCh- and CER-induced amylase secretion. | Linari  *et al*., 2009(Linari et al., 2009) |
| CER (10^-9^ M) | Rat PACs | CB1R, CB2R; pro-inflammatory factors | WIN55,212, 10^-6^ M | CBR agonist reduced IL-6 and MCP-1 in CER-stimulated PACs. | Petrella  *et al*., 2010(Petrella et al., 2010) |
| ACh (10 nM)  CCK (10 pM)  L-Arg (10 mM) | CB2-r knockout and wild type mouse PACs | CB2R; Ca^2+^ oscillations | GW405833 (CB2R agonist)  AM630 | CB2R agonist reduced ACh/L-Arg-, but not CCK-induced Ca^2+^ oscillation, prevented by CB2R antagonist. | Huang  *et al*., 2016(Huang et al., 2016) |
| ACh (10 nM) | Mouse PACs | CB2R; Ca^2+^ oscillations | GW, GP1a, JWH133, SER601, CB65, JWH015, Hu308, L759656 (all CB2R agonists);  ACEA (CB1R agonist);  WIN 55,212;  2-AG (endocannabinoid ligand) | CB2R agonists inhibited ACh-induced Ca^2+^ oscillations and other cannabinoid ligand-modulated Ca^2+^ oscillations in a heterogeneous manner. | Xia  *et al*., 2019(Xia et al., 2019) |

*CBRs, cannabinoid receptors;* *PACs, pancreatic acinar cells; CER, caerulein; CCh, carbachol; KCl, potassium chloride; CB1R, cannabinoid type 1 receptor; CB2R, cannabinoid type 2 receptor; IL-6, interleukin-6; MCP-1, monocyte chemoattractant protein-1; ACh, acetylcholine; CCK, cholecystokinin; L-Arg, L-arginine.*

**Supplementary Table 8 |** *In vivo* studies on CBRs in experimental AP models.

| **AP model** | **Species** | **Pain assessment** | **Mechanism** | **Intervention** | **Effects** | **Ref** |
| --- | --- | --- | --- | --- | --- | --- |
| CER (100 μg/kg, i.v. via right jugular vein)  NaTC (5%, 1 mg/kg, 0.2 mL/min, i.d.) | Rats | NR | CB1R | AM251 (CB1R antagonist), 3 mg/kg, i.v., via the right jugular vein | 1) Plasma anandamide levels were increased in both models with NaTC-AP higher than CER-AP;  2) CB1R antagonism improved the mean arterial pressure and survival rate of NaTC-AP rats. | Matsuda  *et al*., 2005(Matsuda et al., 2005) |
| CER (5 × 50 μg/kg/h, i.p.) | Rats | NR | CB1R | Anandamide (CB1R agonist), 0.8, 1.5, 3 μM/kg, i.p.;  AM251, 4 μM/kg, i.p. | 1) CB1R activation increased the severity of CER-AP;  2) CB1R inhibition reduced pancreatic histopathological and biochemical indices in CER-AP. | Dembiński  *et al*., 2006(Dembinski et al., 2006) |
| CER (10 × 50 μg/kg/h, i.p.) | Mice | Abdomen von Frey test^*^ | CB1R and CB2R | HU210 (nonselective CBR agonist), 0.05 mg/kg, i.p.;  AM251, 3 mg/kg, i.p.;  AM630 (CB2R antagonist), 1 mg/kg, i.p. | CBR agonists reduced pain and pancreatic injury in CER-AP, which was reversed by CBR antagonists | Michalski  *et al*., 2007(Michalski et al., 2007) |
| CER (5 × 50 μg/kg/h, i.p.) | Rats | NR | CB1R | Anandamide, 1.5 μmol/kg, i.p.;  Capsaicin (sensory nerve stimulant), 0.5 mg/kg, s.c. | 1) Pre-treatment with CB1R agonist increased, while sensory nerve stimulant reduced CER-AP severity which was abolished by co-current CB1R agonist;  2) Post-treatment with CB1R agonist and sensory nerve stimulant showed opposite effects | Dembiński  et al., 2008(Dembinski et al., 2008) |
| CER (3 × 10 μg/kg/h, i.p.) | Rats | NR | CB1R and CB2R | / | CB1R expression was not significantly changed;  CB2R expression is reduced in CER-AP | Linari  *et al*., 2009(Linari et al., 2009) |
| CER (6 × 50 μg/kg/h, i.p.) | Mice | NR | CB1R | Rimonabant (CB1R antagonist), 10 mg/kg, i.p., injection daily for 7 days | CB1R antagonism alleviated CER-AP severity in obese mice, partly mediated by increasing circulating anti-inflammatory adipokine adiponectin levels | Zyromski  et al., 2009(Zyromski et al., 2009) |
| CER (3 × 10 μg/kg/h, i.p.) | Rats | NR | CB1R and CB2R | WIN 55,212 (nonselective CBR agonist), 2 mg/kg, i.p.;  AM630 (CB2R specific antagonist), 2 mg/ kg, i.p.;  AM251 (CB1R specific antagonist), 2 mg/kg, i.p. | Pre-treatment with CBR agonist reduced IL-6, MCP-1, and severity of CER-AP, while post-treatment showed converse effects | Petrella  *et al*., 2010(Petrella et al., 2010) |
| CER (6 × 50 μg/kg/h, i.p.) | Mice | NR | CB2R-MAPK/MK2-AP attenuation | CB1R and MK2 knockout;  HU210 (nonselective CBR agonist), 50 μg/kg, i.p.;  JWH133 (CB2R agonist), 5 mg/kg, i.p.;  AM281 (CB1R antagonist), 1 mg/kg, i.p.;  AM630 (CB2R antagonist), 1 mg/kg, i.p. | CB2R activation reduced CER-AP severity via MAPK/MK2-dependent pathway | Michler *et al*., 2013(Michler et al., 2013) |
| L-Arg (4.0 g/kg, i.p.) | Mice | NR | CB2R | GW405833 (CB2R agonist), 10 mg/kg, i.p. | CB2R activation alleviated ARG-AP | Huang  *et al*., 2016(Huang et al., 2016) |
| CER (10 × 50 μg/kg/h, i.p.) | Mice | Abdomen von Frey test^#^, 11 and 12 h after the first dose of CER | CB2R | TPL-Xa (heteropolysaccharide), 10 mg/kg, i.v.;  AM281 (CB1R antagonist), 3 mg/kg, s.c.;  AM630 (CB2R antagonist), 1 mg/kg, s.c. | TPL-Xa inhibited hyper-nociception and inflammation in CER-AP *via* CB2R involved mechanism | Silva-Leite  *et al*., 2018(da Silva-Leite et al., 2018) |

^*^The von Frey filaments (0.008g to 0.6g) were used to examine the frequency of abdominal nocifensive reactions (abdominal licking, abdominal and/or whole-body withdrawal) in response to graded punctate abdominal pressure. The average number of withdrawals observed from 10 applications of the relevant filament spaced 10 seconds apart was used to calculated withdrawal frequency. ^#^Mice were put in a transparent cage with high wire mesh platforms. The abdominal hypernociceptive reflex (licking of the belly, abdominal and/or whole-body withdrawal) was elicited by applying a gradual pressure (g) with a polypropylene tip (0.5 mm2 contact area) attached to a hand-held force transducer (Electronic von Frey Aesthesiometer). *CBRs, cannabinoid receptors; AP, acute pancreatitis; CER, caerulein; i.v., intravenous; NaTC, sodium taurocholate; i.d., intrapancreatic duct; NR, not reported; CB-r, cannabinoid receptor; CER-AP, caerulein-induced acute pancreatitis; i.p., intraperitoneal; s.c., subcutaneous; PACs, pancreatic acinar cells; IL-6, interleukin-6; MCP-1, monocyte chemoattractant protein-1; i.d., intraductal; MAPK, mitogen-activated protein kinase; MK2, MAPK-activated protein kinase 2; L-Arg, L-arginine; ARG-AP, L-arginine-induced acute pancreatitis;* *TPL-Xa, total polysaccharide of X.* *americana.*

Amiti, Tamizhselvi, R., and Manickam, V. (2019). Menadione (vitamin K3) inhibits hydrogen sulfide and substance P via NF-кB pathway in caerulein-induced acute pancreatitis and associated lung injury in mice. *Pancreatology* 19(2)**,** 266-273. doi: 10.1016/j.pan.2019.01.012.

Barreto, S.G., Carati, C.J., Schloithe, A.C., Toouli, J., and Saccone, G.T.P. (2010). The combination of neurokinin-1 and galanin receptor antagonists ameliorates caerulein-induced acute pancreatitis in mice. *Peptides* 31(2)**,** 315-321. doi: 10.1016/j.peptides.2009.11.014.

Bhatia, M., Saluja, A.K., Hofbauer, B., Frossard, J.L., Lee, H.S., Castagliuolo, I., et al. (1998). Role of substance P and the neurokinin 1 receptor in acute pancreatitis and pancreatitis-associated lung injury. *Proceedings of the National Academy of Sciences of the United States of America* 95(8)**,** 4760-4765.

Bhatia, M., Sidhapuriwala, J.N., Wei Ng, S., Tamizhselvi, R., and Moochhala, S.M. (2008). Pro-inflammatory effects of hydrogen sulphide on substance P in caerulein-induced acute pancreatitis. *Journal of Cellular and Molecular Medicine* 12(2)**,** 580-590.

Camargo, E.A., Ferreira, T., Ribela, M.T., de Nucci, G., Landucci, E.C., and Antunes, E. (2008). Role of substance P and bradykinin in acute pancreatitis induced by secretory phospholipase A2. *Pancreas* 37(1)**,** 50-55. doi: 10.1097/MPA.0b013e3185d9b9b.

Ceppa, E., Cattaruzza, F., Lyo, V., Amadesi, S., Pelayo, J.C., Poole, D.P., et al. (2010). Transient receptor potential ion channels V4 and A1 contribute to pancreatitis pain in mice. *Am J Physiol Gastrointest Liver Physiol* 299(3)**,** G556-571. doi: 10.1152/ajpgi.00433.2009.

da Silva-Leite, K.E.S., Girão, D., de Freitas Pires, A., Assreuy, A.M.S., de Moraes, P.A.F., Cunha, A.P., et al. (2018). Ximenia americana heteropolysaccharides ameliorate inflammation and visceral hypernociception in murine caerulein-induced acute pancreatitis: Involvement of CB2 receptors. *Biomed Pharmacother* 106**,** 1317-1324. doi: 10.1016/j.biopha.2018.07.067.

Dembinski, A., Warzecha, Z., Ceranowicz, P., Dembinski, M., Cieszkowski, J., Pawlik, W.W., et al. (2006). Cannabinoids in acute gastric damage and pancreatitis. *Journal of physiology and pharmacology : an official journal of the Polish Physiological Society* 57 Suppl 5**,** 137-154.

Dembiński, A., Warzecha, Z., Ceranowicz, P., Jaworek, J., Sendur, R., Knafel, A., et al. (2003). Stimulation of sensory nerves and CGRP attenuate pancreatic damage in ischemia/reperfusion induced pancreatitis. *Med Sci Monit* 9(12)**,** Br418-425.

Dembinski, A., Warzecha, Z., Ceranowicz, P., Warzecha, A.M., Pawlik, W.W., Dembinski, M., et al. (2008). Dual, time-dependent deleterious and protective effect of anandamide on the course of cerulein-induced acute pancreatitis. Role of sensory nerves. *European journal of pharmacology* 591(1-3)**,** 284-292. doi: 10.1016/j.ejphar.2008.06.059.

Grady, E.F., Yoshimi, S.K., Maa, J., Valeroso, D., Vartanian, R.K., Rahim, S., et al. (2000). Substance P mediates inflammatory oedema in acute pancreatitis via activation of the neurokinin-1 receptor in rats and mice. *British Journal of Pharmacology* 130(3)**,** 505-512.

Han, C., Du, D., Wen, Y., Li, J., Wang, R., Jin, T., et al. (2021). Chaiqin chengqi decoction ameliorates acute pancreatitis in mice via inhibition of neuron activation-mediated acinar cell SP/NK1R signaling pathways. *J Ethnopharmacol* 274**,** 114029. doi: 10.1016/j.jep.2021.114029.

He, Z.J., Winston, J.H., Yusuf, T.E., Micci, M.A., Elfert, A., Xiao, S.Y., et al. (2003). Intraductal administration of an NK1 receptor antagonist attenuates the inflammatory response to retrograde infusion of radiological contrast in rats: implications for the pathogenesis and prevention of ERCP-induced pancreatitis. *Pancreas* 27(1)**,** e13-17. doi: 10.1097/00006676-200307000-00018.

Huang, H., Wang, M., Guo, Z., Wu, D., Wang, H., Jia, Y., et al. (2021). Rutaecarpine alleviates acute pancreatitis in mice and AR42J cells by suppressing the MAPK and NF-κB signaling pathways via calcitonin gene-related peptide. *Phytother Res* 35(11)**,** 6472-6485. doi: 10.1002/ptr.7301.

Huang, Z., Wang, H., Wang, J., Zhao, M., Sun, N., Sun, F., et al. (2016). Cannabinoid receptor subtype 2 (CB2R) agonist, GW405833 reduces agonist-induced Ca(2+) oscillations in mouse pancreatic acinar cells. *Sci Rep* 6**,** 29757. doi: 10.1038/srep29757.

Hutter, M.M., Wick, E.C., Day, A.L., Maa, J., Zerega, E.C., Richmond, A.C., et al. (2005). Transient receptor potential vanilloid (TRPV-1) promotes neurogenic inflammation in the pancreas via activation of the neurokinin-1 receptor (NK-1R). *Pancreas* 30(3)**,** 260-265. doi: 10.1097/01.mpa.0000153616.63384.24.

Jochheim, L.S., Odysseos, G., Hidalgo-Sastre, A., Zhong, S., Staufer, L.M., Kroiss, M., et al. (2019). The neuropeptide receptor subunit RAMP1 constrains the innate immune response during acute pancreatitis in mice. *Pancreatology* 19(4)**,** 541-547. doi: 10.1016/j.pan.2019.05.455.

Kanju, P., Chen, Y., Lee, W., Yeo, M., Lee, S.H., Romac, J., et al. (2016). Small molecule dual-inhibitors of TRPV4 and TRPA1 for attenuation of inflammation and pain. *Sci Rep* 6**,** 26894. doi: 10.1038/srep26894.

Koh, Y.-H., Tamizhselvi, R., and Bhatia, M. (2010). Extracellular signal-regulated kinase 1/2 and c-Jun NH2-terminal kinase, through nuclear factor-kappaB and activator protein-1, contribute to caerulein-induced expression of substance P and neurokinin-1 receptors in pancreatic acinar cells. *The Journal of pharmacology and experimental therapeutics* 332(3)**,** 940-948. doi: 10.1124/jpet.109.160416.

Koh, Y.-H., Tamizhselvi, R., Moochhala, S., Bian, J.-S., and Bhatia, M. (2011a). Role of protein kinase C in caerulein induced expression of substance P and neurokinin-1-receptors in murine pancreatic acinar cells. *Journal of cellular and molecular medicine* 15(10)**,** 2139-2149. doi: 10.1111/j.1582-4934.2010.01205.x.

Koh, Y.H., Moochhala, S., and Bhatia, M. (2011b). The role of neutral endopeptidase in caerulein-induced acute pancreatitis. *Journal of Immunology* 187(10)**,** 5429-5439.

Koh, Y.H., Moochhala, S., and Bhatia, M. (2012). Activation of neurokinin-1 receptors up-regulates substance P and neurokinin-1 receptor expression in murine pancreatic acinar cells. *Journal of Cellular and Molecular Medicine* 16(7)**,** 1582-1592.

Lau, H.Y., and Bhatia, M. (2006). The effect of CP96,345 on the expression of tachykinins and neurokinin receptors in acute pancreatitis. *Journal of Pathology* 208(3)**,** 364-371.

Lau, H.Y., Wong, F.L., and Bhatia, M. (2005). A key role of neurokinin 1 receptors in acute pancreatitis and associated lung injury. *Biochem Biophys Res Commun* 327(2)**,** 509-515. doi: 10.1016/j.bbrc.2004.12.030.

Li, B., Han, X., Ye, X., Ni, J., Wu, J., Dai, J., et al. (2018). Substance P-regulated leukotriene B4 production promotes acute pancreatitis-associated lung injury through neutrophil reverse migration. *International Immunopharmacology* 57**,** 147-156.

Linari, G., Agostini, S., Amadoro, G., Ciotti, M.T., Florenzano, F., Improta, G., et al. (2009). Involvement of cannabinoid CB1- and CB2-receptors in the modulation of exocrine pancreatic secretion. *Pharmacol Res* 59(3)**,** 207-214. doi: 10.1016/j.phrs.2008.11.002.

Maa, J., Grady, E.F., Yoshimi, S.K., Drasin, T.E., Kim, E.H., Hutter, M.M., et al. (2000). Substance P is a determinant of lethality in diet-induced hemorrhagic pancreatitis in mice. *Surgery* 128(2)**,** 232-239. doi: 10.1067/msy.2000.107378.

Mathan Kumar, M., and Tamizhselvi, R. (2020). Protective effect of diallyl disulfide against cerulein-induced acute pancreatitis and associated lung injury in mice. *International Immunopharmacology* 80 (no pagination)(106136).

Matsuda, K., Mikami, Y., Takeda, K., Fukuyama, S., Egawa, S., Sunamura, M., et al. (2005). The cannabinoid 1 receptor antagonist, AM251, prolongs the survival of rats with severe acute pancreatitis. *Tohoku J Exp Med* 207(2)**,** 99-107. doi: 10.1620/tjem.207.99.

Michalski, C.W., Laukert, T., Sauliunaite, D., Pacher, P., Bergmann, F., Agarwal, N., et al. (2007). Cannabinoids ameliorate pain and reduce disease pathology in cerulein-induced acute pancreatitis. *Gastroenterology* 132(5)**,** 1968-1978. doi: 10.1053/j.gastro.2007.02.035.

Michler, T., Storr, M., Kramer, J., Ochs, S., Malo, A., Reu, S., et al. (2013). Activation of cannabinoid receptor 2 reduces inflammation in acute experimental pancreatitis via intra-acinar activation of p38 and MK2-dependent mechanisms. *American Journal of Physiology - Gastrointestinal and Liver Physiology* 304(2)**,** G181-G192.

Nathan, J.D., Patel, A.A., McVey, D.C., Thomas, J.E., Prpic, V., Vigna, S.R., et al. (2001). Capsaicin vanilloid receptor-1 mediates substance P release in experimental pancreatitis. *Am J Physiol Gastrointest Liver Physiol* 281(5)**,** G1322-1328. doi: 10.1152/ajpgi.2001.281.5.G1322.

Nishimura, S., Ishikura, H., Matsunami, M., Shinozaki, Y., Sekiguchi, F., Naruse, M., et al. (2010). The proteinase/proteinase-activated receptor-2/transient receptor potential vanilloid-1 cascade impacts pancreatic pain in mice. *Life Sci* 87(19-22)**,** 643-650. doi: 10.1016/j.lfs.2010.09.030.

Noble, M.D., Romac, J., Vigna, S.R., and Liddle, R.A. (2008). A pH-sensitive, neurogenic pathway mediates disease severity in a model of post-ERCP pancreatitis. *Gut* 57(11)**,** 1566-1571. doi: 10.1136/gut.2008.148551.

Noble, M.D., Romac, J., Wang, Y., Hsu, J., Humphrey, J.E., and Liddle, R.A. (2006). Local disruption of the celiac ganglion inhibits substance P release and ameliorates caerulein-induced pancreatitis in rats. *Am J Physiol Gastrointest Liver Physiol* 291(1)**,** G128-134. doi: 10.1152/ajpgi.00442.2005.

Petrella, C., Agostini, S., Alema, G.S., Casolini, P., Carpino, F., Giuli, C., et al. (2010). Cannabinoid agonist WIN55,212 in vitro inhibits interleukin-6 (IL-6) and monocyte chemo-attractant protein-1 (MCP-1) release by rat pancreatic acini and in vivo induces dual effects on the course of acute pancreatitis. *Neurogastroenterol Motil* 22(11)**,** 1248-1256, e1323. doi: 10.1111/j.1365-2982.2010.01569.x.

Ramnath, R.D., and Bhatia, M. (2006). Substance P treatment stimulates chemokine synthesis in pancreatic acinar cells via the activation of NF-kappaB. *American Journal of Physiology - Gastrointestinal and Liver Physiology* 291(6)**,** G1113-G1119.

Ramnath, R.D., Sun, J., and Bhatia, M. (2009). Involvement of Src family kinases in substance P-induced chemokine production in mouse pancreatic acinar cells and its significance in acute pancreatitis. *Journal of Pharmacology and Experimental Therapeutics* 329(2)**,** 418-428.

Romac, J.M., McCall, S.J., Humphrey, J.E., Heo, J., and Liddle, R.A. (2008). Pharmacologic disruption of TRPV1-expressing primary sensory neurons but not genetic deletion of TRPV1 protects mice against pancreatitis. *Pancreas* 36(4)**,** 394-401. doi: 10.1097/MPA.0b013e318160222a.

Schneider, L., Hartwig, W., Flemming, T., Hackert, T., Fortunato, F., Heck, M., et al. (2009). Protective effects and anti-inflammatory pathways of exogenous calcitonin gene-related peptide in severe necrotizing pancreatitis. *Pancreatology : official journal of the International Association of Pancreatology (IAP) ... [et al.]* 9(5)**,** 662-669. doi: 10.1159/000212099.

Schwartz, E.S., Christianson, J.A., Chen, X., La, J.H., Davis, B.M., Albers, K.M., et al. (2011). Synergistic role of TRPV1 and TRPA1 in pancreatic pain and inflammation. *Gastroenterology* 140(4)**,** 1283-1291.e1281-1282. doi: 10.1053/j.gastro.2010.12.033.

Schwartz, E.S., La, J.-H., Scheff, N.N., Davis, B.M., Albers, K.M., and Gebhart, G.F. (2013). TRPV1 and TRPA1 antagonists prevent the transition of acute to chronic inflammation and pain in chronic pancreatitis. *The Journal of neuroscience : the official journal of the Society for Neuroscience* 33(13)**,** 5603-5611. doi: 10.1523/JNEUROSCI.1806-12.2013.

Shahid, R.A., Vigna, S.R., Layne, A.C., Romac, J.M.J., and Liddle, R.A. (2015). Acinar Cell Production of Leukotriene B-4 Contributes to Development of Neurogenic Pancreatitis in Mice. *Cellular and Molecular Gastroenterology and Hepatology* 1(1)**,** 75-86. doi: 10.1016/j.jcmgh.2014.11.002.

Shi, X., Gao, N.R., Guo, Q.M., Yang, Y.J., Huo, M.D., Hu, H.L., et al. (2003). Relationship between overexpression of NK-1R, NK-2R and intestinal mucosal damage in acute necrotizing pancreatitis. *World J Gastroenterol* 9(1)**,** 160-164. doi: 10.3748/wjg.v9.i1.160.

Sun, J., and Bhatia, M. (2007). Blockade of neurokinin-1 receptor attenuates CC and CXC chemokine production in experimental acute pancreatitis and associated lung injury. *American Journal of Physiology - Gastrointestinal and Liver Physiology* 292(1)**,** G143-G153.

Swain, S.M., Romac, J.M., Shahid, R.A., Pandol, S.J., Liedtke, W., Vigna, S.R., et al. (2020). TRPV4 channel opening mediates pressure-induced pancreatitis initiated by Piezo1 activation. *J Clin Invest* 130(5)**,** 2527-2541. doi: 10.1172/jci134111.

Tamizhselvi, R., Moore, P.K., and Bhatia, M. (2007). Hydrogen sulfide acts as a mediator of inflammation in acute pancreatitis: in vitro studies using isolated mouse pancreatic acinar cells. *J Cell Mol Med* 11(2)**,** 315-326. doi: 10.1111/j.1582-4934.2007.00024.x.

Terada, Y., Fujimura, M., Nishimura, S., Tsubota, M., Sekiguchi, F., and Kawabata, A. (2015). Roles of Cav3.2 and TRPA1 channels targeted by hydrogen sulfide in pancreatic nociceptive processing in mice with or without acute pancreatitis. *J Neurosci Res* 93(2)**,** 361-369. doi: 10.1002/jnr.23490.

Terada, Y., Fujimura, M., Nishimura, S., Tsubota, M., Sekiguchi, F., Nishikawa, H., et al. (2013). Contribution of TRPA1 as a downstream signal of proteinase-activated receptor-2 to pancreatic pain. *J Pharmacol Sci* 123(3)**,** 284-287. doi: 10.1254/jphs.13128sc.

Terada, Y., Tsubota, M., Sugo, H., Wakitani, K., Sekiguchi, F., Wada, K., et al. (2017). Tacrolimus Triggers Transient Receptor Potential Vanilloid-1-Dependent Relapse of Pancreatitis-Related Pain in Mice. *Pharmacology* 99(5-6)**,** 281-285. doi: 10.1159/000454816.

Tsukamoto, A., Ohgoda, M., Haruki, N., Hori, M., and Inomata, T. (2018). The anti-inflammatory action of maropitant in a mouse model of acute pancreatitis. *The Journal of veterinary medical science* 80(3)**,** 492-498.

Vera-Portocarrero, L.P., and Westlund, K.N. (2004). Attenuation of nociception in a model of acute pancreatitis by an NK-1 antagonist. *Pharmacol Biochem Behav* 77(3)**,** 631-640. doi: 10.1016/j.pbb.2004.01.004.

Vigna, S.R., Shahid, R.A., and Liddle, R.A. (2014). Ethanol contributes to neurogenic pancreatitis by activation of TRPV1. *Faseb j* 28(2)**,** 891-896. doi: 10.1096/fj.13-236208.

Vigna, S.R., Shahid, R.A., Nathan, J.D., McVey, D.C., and Liddle, R.A. (2011). Leukotriene B4 mediates inflammation via TRPV1 in duct obstruction-induced pancreatitis in rats. *Pancreas* 40(5)**,** 708-714. doi: 10.1097/MPA.0b013e318214c8df.

Wang, T., Jiang, L., Wei, X., Liu, B., Zhao, J., Xie, P., et al. (2018). MiR-21-3p aggravates injury in rats with acute hemorrhagic necrotizing pancreatitis by activating TRP signaling pathway. *Biomed Pharmacother* 107**,** 1744-1753. doi: 10.1016/j.biopha.2018.08.164.

Warzecha, Z., Dembiński, A., Ceranowicz, P., Konturek, P.C., Niemiec, J., Stachura, J., et al. (2000). The influence of sensory nerves and CGRP on the pancreatic regeneration after repeated episodes of acute pancreatitis in rats. *J Physiol Pharmacol* 51(3)**,** 449-461.

Warzecha, Z., Dembiński, A., Ceranowicz, P., Konturek, P.C., Stachura, J., Konturek, S.J., et al. (1997). Protective effect of calcitonin gene-related peptide against caerulein-induced pancreatitis in rats. *J Physiol Pharmacol* 48(4)**,** 775-787.

Warzecha, Z., Dembiński, A., Ceranowicz, P., Konturek, P.C., Stachura, J., Tomaszewska, R., et al. (1999). Calcitonin gene-related peptide can attenuate or augment pancreatic damage in caerulein-induced pancreatitis in rats. *J Physiol Pharmacol* 50(1)**,** 49-62.

Warzecha, Z., Dembiński, A., Ceranowicz, P., Stachura, J., Tomaszewska, R., and Konturek, S.J. (2001). Effect of sensory nerves and CGRP on the development of caerulein-induced pancreatitis and pancreatic recovery. *J Physiol Pharmacol* 52(4 Pt 1)**,** 679-704.

Wick, E.C., Hoge, S.G., Grahn, S.W., Kim, E., Divino, L.A., Grady, E.F., et al. (2006a). Transient receptor potential vanilloid 1, calcitonin gene-related peptide, and substance P mediate nociception in acute pancreatitis. *Am J Physiol Gastrointest Liver Physiol* 290(5)**,** G959-969. doi: 10.1152/ajpgi.00154.2005.

Wick, E.C., Pikios, S., Grady, E.F., and Kirkwood, K.S. (2006b). Calcitonin gene-related peptide partially mediates nociception in acute experimental pancreatitis. *Surgery* 139(2)**,** 197-201. doi: 10.1016/j.surg.2005.08.024.

Xia, K.K., Shen, J.X., Huang, Z.B., Song, H.M., Gao, M., Chen, D.J., et al. (2019). Heterogeneity of cannabinoid ligand-induced modulations in intracellular Ca(2+) signals of mouse pancreatic acinar cells in vitro. *Acta Pharmacol Sin* 40(3)**,** 410-417. doi: 10.1038/s41401-018-0074-y.

Yan, L., Li, Q.-F., Rong, Y.-T., Chen, Y.-H., Huang, Z.-H., Wang, Z.-Z., et al. (2018). The protective effects of rutaecarpine on acute pancreatitis. *Oncology letters* 15(3)**,** 3121-3126. doi: 10.3892/ol.2017.7659.

Zyromski, N.J., Mathur, A., Pitt, H.A., Wade, T.E., Wang, S., Swartz-Basile, D.A., et al. (2009). Cannabinoid receptor-1 blockade attenuates acute pancreatitis in obesity by an adiponectin mediated mechanism. *J Gastrointest Surg* 13(5)**,** 831-838. doi: 10.1007/s11605-009-0824-8.
